# Mechanistic insights into electrochemiluminescence: fueling advances in bioanalysis and imaging

**DOI:** 10.1039/d6sc04645b

**Published:** 2026-08-06

**Authors:** Kaiqing Wu, Yuanjian Zhang, Paul S. Francis, Giovanni Valenti, Neso Sojic

**Affiliations:** a Univ. Bordeaux, Bordeaux INP, ISM, UMR CNRS 5255 Pessac 33607 France sojic@u-bordeaux.fr; b Jiangsu Engineering Laboratory of Smart Carbon-Rich Materials and Device, School of Chemistry and Chemical Engineering, Southeast University Nanjing 211189 China; c School of Life and Environmental Sciences, Faculty of Science, Engineering and Built Environment, Deakin University Waurn Ponds Victoria 3216 Australia; d Department of Chemistry “Giacomo Ciamician”, Alma Mater Studiorum – University of Bologna Via Gobetti 85 40129 Bologna Italy

## Abstract

The rational improvement of electrochemiluminescence (ECL) features such as increased signal intensity and both spatial and temporal extension requires deciphering the competitive mechanistic pathways occurring in the vicinity of the electrode surface. This understanding is critical for developing high-performance ECL analytical systems. This perspective systematically reviews the advanced characterization and theoretical simulation techniques for uncovering ECL reaction mechanisms, including ECL microscopy, ECL-scanning electrochemical microscopy, ECL self-interference spectroscopy, electrochemistry-mass spectrometry, and ECL-voltammetric analysis and numerical simulation. Emerging ECL enhancement mechanisms and regulatory pathways are comprehensively elaborated, covering peroxydisulfate-oxalate and redox-mediated mechanisms, time-scale regulation strategies of radical stabilization, time matching, and pre-oxidation/reduction, as well as exogenous stimulation modulation *via* heat, ultrasound, and light. Moreover, this article highlights recent progress in the application of enhanced ECL platforms in bioanalysis and bioimaging. This review offers a systematic and instructive reference for the rational design and further development of high-efficiency ECL systems for advanced bioanalysis and microscopy applications.

## Abstract 2

Emerging mechanistic strategies and advanced mechanism evaluation techniques for fueling advances in bioanalysis and imaging.

## Graphical abstract

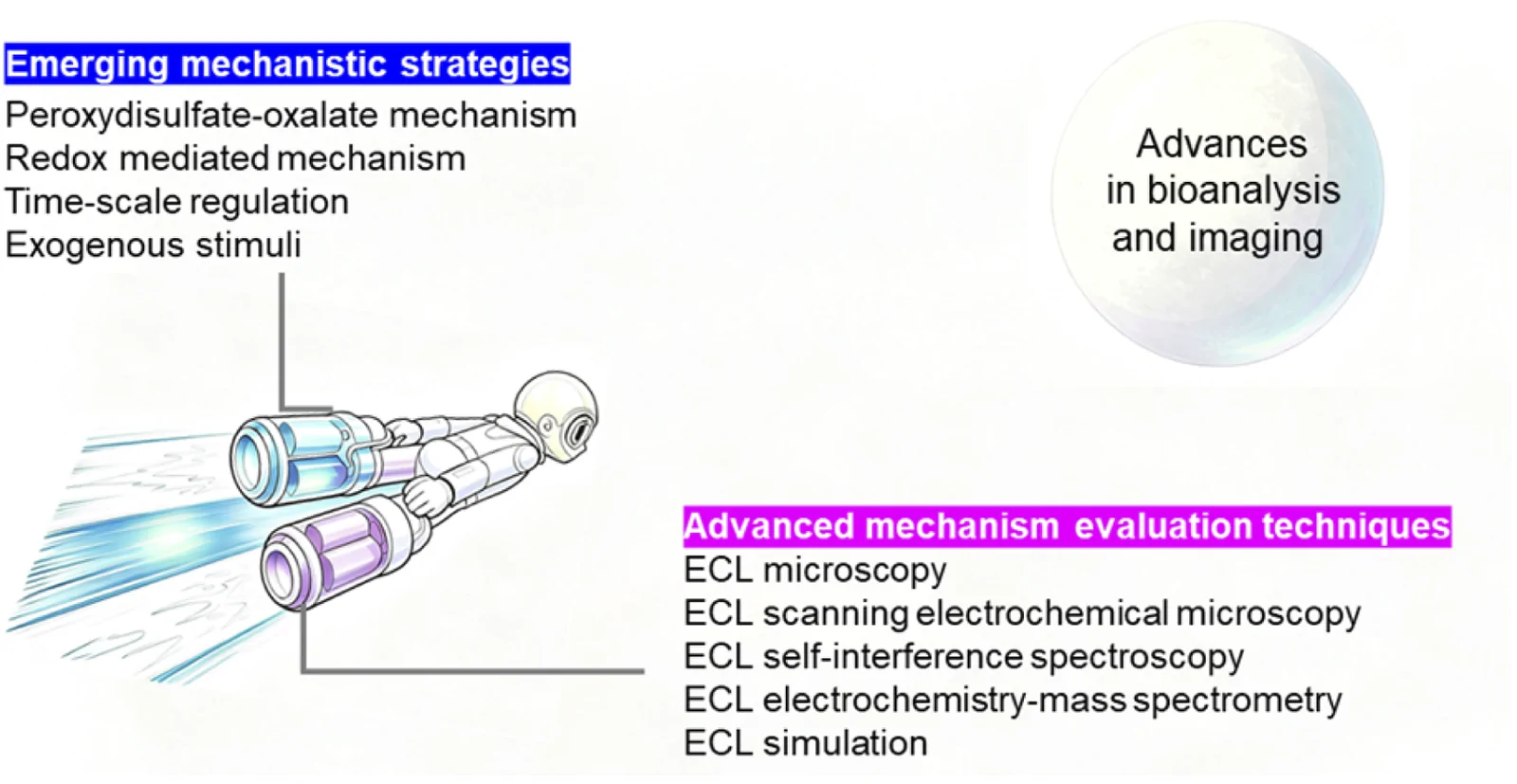

## 1 Introduction

Electrochemiluminescence (ECL) refers to the luminescence phenomenon arising from electrochemical oxidation and reduction of specific substances.^1,2^ In this process, ECL-active species undergo high-energy electron transfer on the electrode surface, leading to the generation of electronically excited intermediates that subsequently return to the ground state *via* radiative relaxation pathways.^3,4^ In recent years, as detection demands have shifted from macroscopic signals to microscale and nanoscale visualization, ECL imaging has gradually enabled the direct observation of single particles, single cells, and interfacial reactions.^5,6^ However, as these high-resolution applications continue to develop, issues of insufficient signal intensity and limited efficiency have become increasingly prominent. Therefore, the achievement of efficient and stable signal enhancement has become a core issue in ECL research.

From the perspective of the reaction nature, the ECL signal originates from a series of consecutive processes, including electrode electron transfer, the generation and diffusion of reactive intermediates, the formation of excited-state species, and the emission of photons.^7–9^ Thermodynamic or kinetic mismatch in any of these processes can decrease the overall ECL efficiency. Therefore, enhancing the ECL signal does not rely on optimizing a single factor, but requires coordinated regulation across multiple aspects, incorporating electron transfer, intermediate generation, mass transport and luminescence efficiencies. To achieve this goal, researchers have gradually established a multidimensional signal enhancement methodology by regulating reaction pathways,^10^ prolonging intermediate lifetimes,^11,12^ and optimizing the reaction microenvironment.^13,14^

In terms of mechanism analysis, advanced characterization techniques and theoretical methods provide critical support for understanding and guiding ECL signal enhancement. For example, electrochemical microscopy can resolve local luminescence behavior in two-dimensional (2D) or three-dimensional (3D) space,^15^ ECL self-interference spectroscopy provides information on the thickness of the luminescent layer (TEL) and the distribution of excited states,^16,17^ and electrochemical-mass spectrometry coupling enables molecular-level capture of short-lived intermediates.^18^ Meanwhile, numerical methods such as finite element simulations can reconstruct reaction kinetics and concentration distributions, providing quantitative bases for parameters that are difficult to measure directly in experiments.^19,20^ The combination of these technical approaches allows ECL signal enhancement to gradually shift from empirical optimization to mechanism-driven rational design.

Based on an in-depth understanding of reaction mechanisms, various emerging ECL signal enhancement pathways have been continuously proposed.^21^ For instance, the peroxydisulfate-oxalate mechanism improves luminescence efficiency by lowering reaction barriers,^22,23^ redox mediators facilitate electron transfer and accelerate reaction kinetics,^24^ and temporal regulation maximizes efficiency by matching the kinetics of different reaction processes.^11,12^ In addition, strategies such as nanoscale confinement effects, self-enhancing systems, micro/nanofluidics,^25^ and external field stimulation further optimize reaction processes from spatial and energy dimensions.^26,27^ These enhancement mechanisms have significantly promoted the application of ECL in highly sensitive bioanalysis and high-resolution imaging, including protein detection, nucleic acid analysis, and single-cell behavior studies. Moreover, measuring ECL efficiency is critical for evaluating and comparing the luminescent performances of materials and reaction systems, serving as a core index to compare the advantages of different enhanced ECL strategies.^28–31^

This perspective, with ECL signal enhancement as the main theme, reviews advanced characterization and theoretical methods used to reveal reaction mechanisms, summarizes emerging enhancement reaction pathways, and discusses their application progress in bioanalysis and bioimaging, aiming to provide a systematic reference for the rational design and future development of high-performance ECL systems.

## 2 Advanced techniques to elucidate ECL mechanisms

### 2.1 Electrochemical microscopy: ECL microscopy (ECLM) and ECL-scanning electrochemical microscopy (ECL-SECM) techniques

**Fig. 1:**
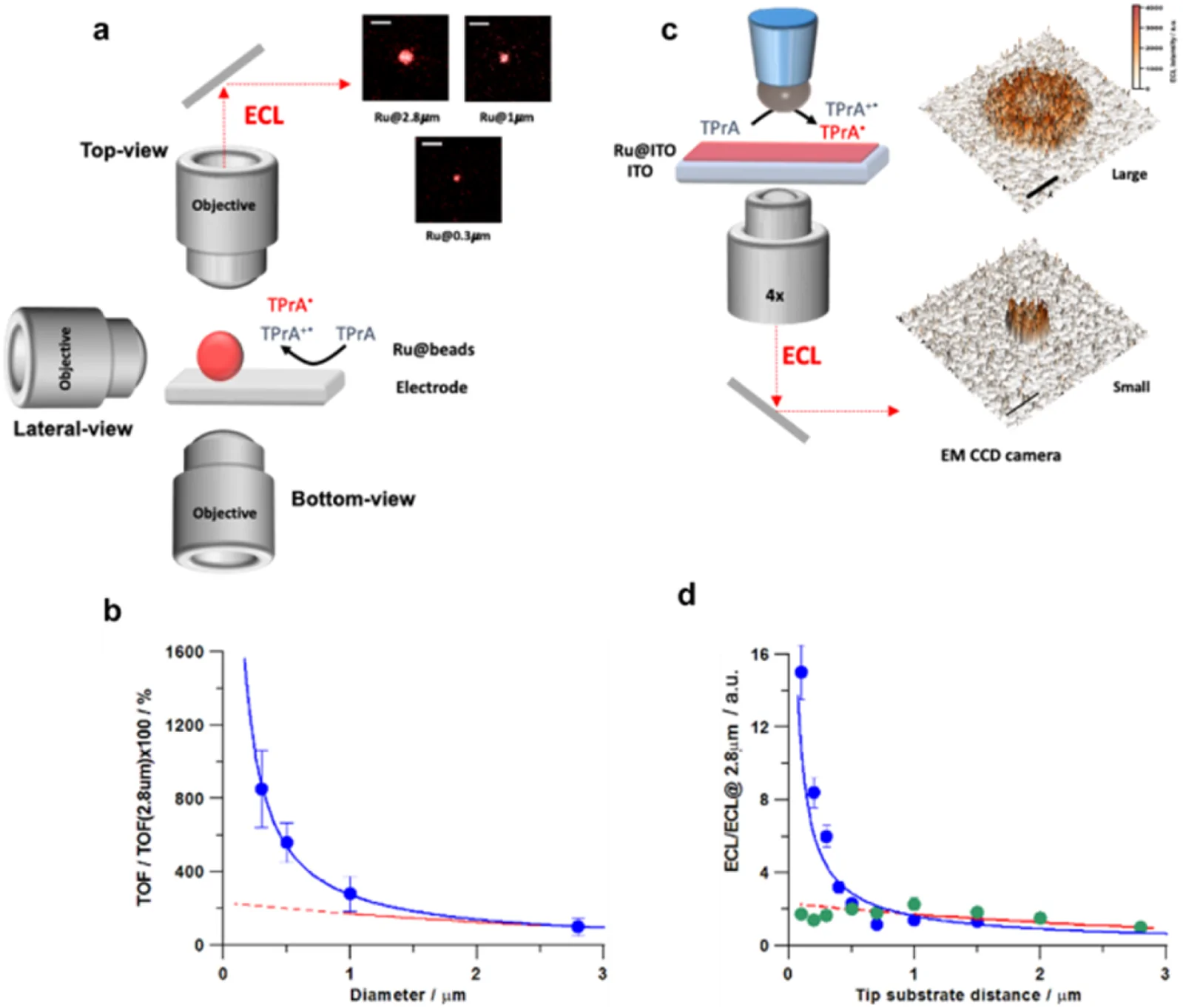
Spatial map of ECL emission. (a) Schematic representation of the surface generation–bead emission experiment where Ru@beads are magnetic beads labelled with [Ru(bpy)_3_]^2+^ on platinum electrode (Pt) and (b) turnover frequency (TOF) as a function of bead size (blue curve and dots) and ECL intensity of the reference (red curve). The inset in (a) shows the ECL images acquired for beads with different sizes where Ru@2.8 µm, Ru@1 µm and Ru@0.3 µm are magnetic beads labelled with [Ru(bpy)_3_]^2+^ with a diameter of 2.8 µm, 1 µm and 0.3 µm respectively. The TOF is the number of photons emitted in 1 s by a single luminophore. (c) Schematic representation of the tip generation–surface emission experiment where Ru@ITO is transparent indium tin oxide (ITO) electrode functionalized with a [Ru(bpy)_3_]^2+^ monolayer as the emitting surface, and (d) ECL intensity as a function of the tip–surface distance for a small electrode (blue curve and dots) and a large electrode (green dots) and ECL intensity of the reference (red curve). ECL@2.8 µm is the ECL intensity for 2.8 µm distance between tip and surface. The inset in (c) shows the ECL intensity image profile acquired for a small and a large Pt hemispherical electrode at 1.4 V (*vs.* Ag/AgCl, 3 M KCl). Adapted from ref. 35 with permission from the Springer Nature, copyright 2020.

Electrochemical microscopy has become a central strategy for elucidating reaction mechanisms because it correlates electrochemical reactivity with spatial resolution.^15^ ECL microscopy (ECLM) maps the light emitted locally during electrochemical reactions at an electrode by combining an electrochemical cell with optical microscopy and charge coupled device (CCD) or electron multiplier CCD (EMCCD) camera detection. Unlike fluorescence microscopy, ECLM requires no external excitation source, which minimizes scattered light and autofluorescence and makes it particularly suitable for bioanalysis.^1,3,32^ These approaches have shown that ECL is intrinsically a surface-confined process whose efficiency depends on the lifetime of co-reactant radicals, the distribution of luminophores, and the TEL. ECL microscopy has become a powerful tool for elucidating ECL mechanisms because it directly visualizes where emission is generated and how the emitting layer evolves near the electrode.^33,34^ In general, the three most used experimental approaches are the (i) top-, (ii) bottom-, and (iii) lateral-view in a so-called surface generation beads emission configuration (Fig. 1a and b). In heterogeneous bead-based systems, ECL luminophores are typically immobilized across the entire surface of microbeads. The beads are micrometric magnetic or polystyrene microspheres whose surfaces are functionalized with tris(2,2′-bipyridine)ruthenium(ii) ([Ru(bpy)_3_]^2+^)-type luminophores, typically through biotin–streptavidin, amide, or immunoassay-based conjugation, so that the ECL labels are distributed over the outer bead surface. These beads have been shown to accurately model heterogeneous bead-based ECL sandwich immunoassays.^35^ Because the luminophores are distributed three-dimensionally on non-conductive microbeads, signal generation in the heterogeneous format depends on the oxidation of sacrificial tertiary amine co-reactants which produce both oxidizing and reducing radical intermediates that are sufficiently long-lived to excite luminophores beyond the immediate vicinity of the electrode surface. In bead-based [Ru(bpy)_3_]^2+^/tri-*n*-propylamine (TPrA) systems, ECL imaging showed that the heterogeneous or “remote” pathway is confined to a thin layer only a few micrometers thick, mainly limited by the short lifetime of TPrA˙^+^ and the requirement that both co-reactant radicals reach immobilized luminophores on the bead surface.^35^ More recent studies further demonstrated that combining ECL microscopy with simulations can disentangle chemical from optical effects, revealing that bead optics strongly shape the recorded signal,^36,37^ and analyze the signal time evolution.^38^

In parallel, scanning electrochemical microscopy (SECM), originally developed as a classical electrochemical imaging method based on an ultramicroelectrode positioned with high precision above a surface, was later coupled with ECL following the pioneering work of Bard and co-workers,^39^ who used ECL generated at an ultramicroelectrode as a localized light source. This coupling enabled ECL-SECM to be used as a mechanistic platform in a so-called tip generation surface emission configuration (Fig. 1c). As the microelectrode can interrogate short-lived intermediates near the substrate, and because the TEL is itself governed by the lifetime and diffusion of reactive radicals, ECL-SECM is especially powerful for probing the spatial organization of ECL pathways. In these experiments, a Pt microelectrode tip was positioned at controlled distances from a [Ru(bpy)_3_]^2+^-modified surface, allowing direct mapping of ECL intensity as a function of distance from the electrode. This strategy demonstrated that the classical remote TPrA pathway does not fully describe heterogeneous ECL close to the surface. At distances larger than about 1 µm, the signal follows the known decay behavior associated with the TPrA radical cation, whose half-life is in the order of hundreds of microseconds. However, below about 1 µm (see blue signal for small tips and green signal for large tips in Fig. 1d), an alternative, much more efficient pathway emerges, characterized by a much faster decay and by ECL intensities around one order of magnitude higher than those observed farther from the interface.^35^ These findings establish ECLM and ECL-SECM not simply as imaging tools, but as mechanistic techniques capable of resolving short-range radical chemistry, identifying parallel reaction channels, and guiding the design of improved bioanalytical systems.

### 2.2 ECL self-interference spectroscopy technique

The ECL generated in the immediate vicinity of the electrode surface is characterized by the TEL, which is related to the lifetimes and diffusion coefficients of the luminescent and co-reactant intermediates. Spatially resolved measurements can directly obtain the distribution of excited state (such as [Ru(bpy)_3_]^2+^*) near the electrode surface or co-reactant radicals, thereby facilitating a deeper understanding of the luminescence mechanism within the [Ru(bpy)_3_]^2+^ system.^32^

**Fig. 2:**
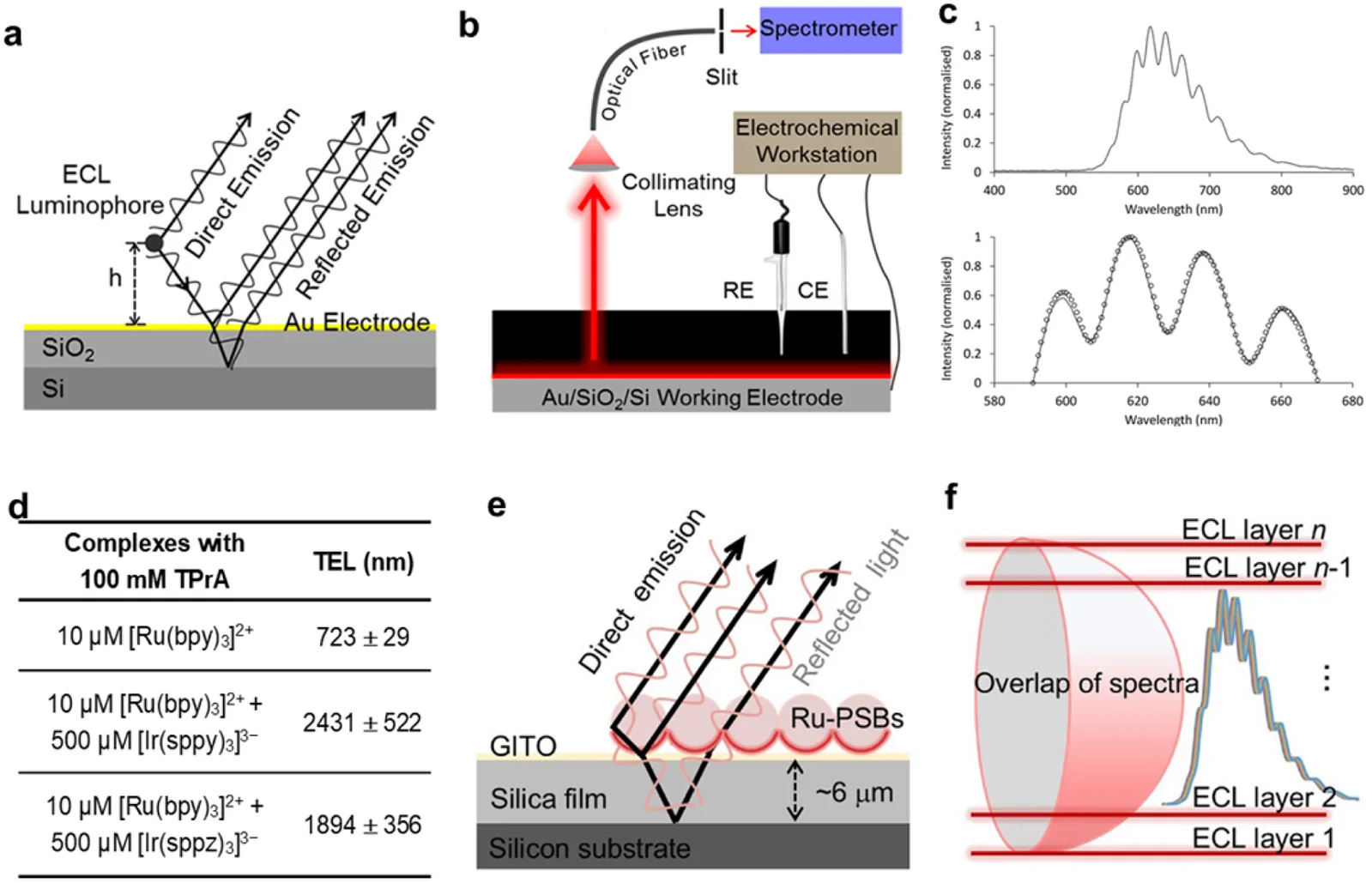
(a) Principle of ECLIS at a gold/silica/silicon electrode. (b) Experimental setup for collecting the ECL self-interference spectrum. Adapted from ref. 40 with permission from the *American Chemical Society*, copyright 2019. (c) Normalized ECLIS spectrum (top) and the corresponding simulation curve (bottom). (d) Thickness of the [Ru(bpy)_3_]^2+^ ECL layer without and with redox mediators ([Ir(sppy)_3_]^3−^ and [Ir(sppz)_3_]^3−^) in solution. Adapted from ref. 43 with permission from the John Wiley and Sons, copyright 2024. (e) Schematic of the ECL self-interference for Ru-PSBs at GITO/silica/silicon electrode. (f) Schematic of calculating the ECL-emitting layer thickness on a single Ru-PSB. Adapted from ref. 41 with permission from the John Wiley and Sons, copyright 2025.

ECL self-interference spectroscopy (ECLIS) was proposed by Su's group.^40^ The technique possessed spatial resolution and allowed the measurement of the vertical distribution of ECL intensity near the electrode surface, and provided valuable insights into reaction mechanisms. The principle of ECLIS is shown in Fig. 2a. The phenomenon of ECLIS is attributed to the superposition effect, which occurs between the light directly emitted by the luminophores and the light that is reflected back from the multilayer interfaces of the gold/silicon/silica electrode. The interference light, once focused by a collimating lens, traverses an optical fiber and slit before being detected by a spectrophotometer (Fig. 2b). Unlike traditional ECL spectra, ECLIS display a series of regularly spaced sharp peaks. These peaks essentially convey spatial position information about the luminophore relative to the electrode surface. By optimally aligning the experimental spectra with theoretical spectra, calculated using the light propagation matrix and optical interference model, the TEL value can be determined. This technology is widely applied in the study of the performance of transparent substrate materials, the measurement of ECL distance and lifetime of co-reactant radical cations.^41–43^

Considering both the activity and lifetime of co-reactants helps in selecting the most suitable for the intended application. Su *et al.* compared various co-reactants with [Ru(bpy)_3_]^2+^, including TPrA, 2-(dibutylamino)ethanol (DBAE), 2,2′-(butylimino)diethanol (BIDE), triethanolamine (TEOA) and 2,2-bis(hydroxymethyl)-2,2′,2″-nitrilotriethanol (BIS-TRIS).^42^ Using ECL intensity (the peak value) as an evaluation parameter, BIDE showed the highest reactivity among all the tested tertiary amines. However, based on ECL imaging analysis, the ECL emission area of [Ru(bpy)_3_]^2+^-labeled polystyrene beads (Ru-PSB) was largest when adopting BIS-TRIS among the five co-reactants. Thus, the lifetimes of DBAE˙^+^, BIDE˙^+^, TEOA˙^+^ and BIS-TRIS˙^+^ were calculated from the ECLIS spectra: *ca.* 58, 18, 5 and 328 µs, respectively, using that of TPrA˙^+^ (200 µs) as the standard. Considering that the reactivity of BIS-TRIS is lower than that of DBAE or BIDE, thus, the trade-off of ECL lifetime-reactivity makes BIS-TRIS potentially a more effective ECL co-reactant for imaging detection, especially when the magnetic beads are smaller.

As discussed in Section 3.2, redox mediators can be used to enhance ECL intensity by promoting co-reactant oxidation, and providing a more stable alternative to the initial oxidation product of the co-reactant for the oxidative excitation of the luminophore. To identify the ECL enhancement pathway of the non-emissive enhancer, sulfonated tris(1-phenylpyrazolato)iridium(iii) ([Ir(sppz)_3_]^3−^), in the [Ru(bpy)_3_]^2+^/TPrA system, an ITO/SiO_2_/Si multilayer electrode to generate ECL self-interference was employed.^43^ Analysis of the interference spectra (Fig. 2c) for the unenhanced and enhanced systems showed that this redox mediator can significantly extend the diffusion distance of active intermediates generated from the oxidation of TPrA from the electrode surface, thereby enhancing the ECL intensity of [Ru(bpy)_3_]^2+^ (Fig. 2d).

The low electrochemical activity of the indium tin oxide (ITO) electrode results in weak ECL signals, while the poor transparency of the Au and Si electrode causes significant light attenuation due to reflection, thereby hindering the acquisition of a clear ECLIS spectrum from the microbeads. To avoid this, a high electrochemical activity and transparency of single-layer graphene-modified ITO (GITO) electrodes was designed (Fig. 2e and f), and the thickness and position of the effective ECL emission layer on microsphere surfaces were successfully measured with ECLIS technology.^41^

### 2.3 Electrochemistry-mass spectrometry (EC-MS)

Capturing short-lived intermediates is essential to understand the mechanism and dynamics of (electro)chemical reactions.^18^ Mass spectrometry (MS) offers extremely high sensitivity and specificity, providing direct information on the composition and structure of various target analytes at the molecular level.^44,45^

**Fig. 3:**
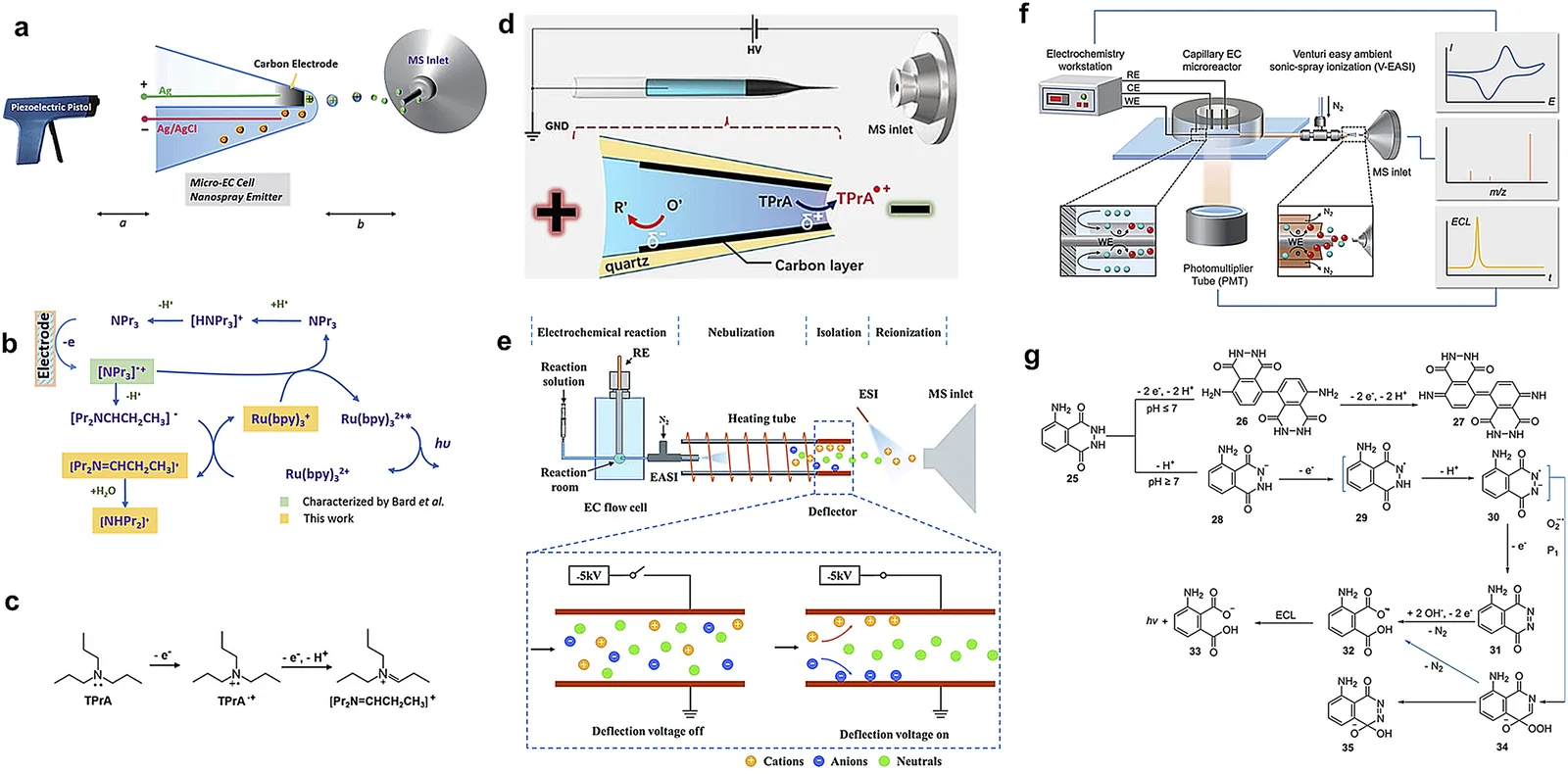
(a) Schematic representation of the EC-*n*ESI-MS device with fabricated carbon electrode and a piezoelectric pistol. (b) Mechanistic route of [Ru(bpy)_3_]^2+^ ECL when a potential of 0.8 V was applied. (c) The proposed electrochemical oxidation process of TPrA. Adapted from ref. 46 with permission from the *Royal Society of Chemistry*, copyright 2016. (d) Schematic representation of the implementation of nESI-MS to detect electrogenerated intermediate. Adapted from ref. 44 with permission from the John Wiley and Sons, copyright 2020. (e) Schematic representation of the EC-NR-MS device assembled with heating tube and deflector for detection of neural radicals. Adapted from ref. 49 with permission from the *Royal Society of Chemistry*, copyright 2021. (f) Schematic representation of the ECL-MS device utilizing electrochemistry workstation and photomultiplier tube. (g) The proposed ECL pathways of luminol depending on the pH. Adapted from ref. 47 with permission from the *Royal Society of Chemistry*, copyright 2022.

Over the past few years, combining electrochemistry with MS gave mass fragmentation patterns of ECL reactions that were valuable in the understanding of ECL mechanistic pathways.^35,46–48^ [Ru(bpy)_3_]^2+^/TPrA is a model co-reactant-ECL system, and its reaction mechanism may follow several competitive routes. To confirm the proposed mechanism using EC-MS,^46^ a novel hybrid ultramicroelectrode comprising a miniature carbon electrode and a hollow microchannel, was developed. This unique configuration allows it to function as both a microelectrochemical cell and a nanospray emitter for MS analysis (Fig. 3a and b). When a potential of 0.8 V *vs.* Ag/AgCl was applied, intermediate cations such as [Pr_2_N=CHCH_2_CH_3_]^+^, [NHPr_2_]^+^, and [Ru(bpy)_3_]^+^ were successfully detected, while no information on [Ru(bpy)_3_]^3+^ was observed. With higher potentials where oxidation of [Ru(bpy)_3_]^2+^ occurs, [Ru(bpy)_3_]^3+^ was detected, while [Ru(bpy)_3_]^+^ was absent. This study not only verified the main mechanistic pathways in the ECL mechanism of the [Ru(bpy)_3_]^2+^/TPrA system but also demonstrated the potential of the new EC-MS device for the investigation of other complicated electrochemical reaction mechanisms. Notably, this work lacked information on TPrA˙^+^, which is generated from TPrA during the process (Fig. 3c). For this, Xu *et al.*^44^ integrated a bipolar ultramicroelectrode with nano-electrospray ionization mass spectrometry (nESI-MS, Fig. 3d), achieving microsecond transfer of transient species from the electrode–electrolyte interface of the ultramicroelectrode into the gas phase for subsequent MS analysis. This high temporal resolution enabled successful capture of the short-lived TPrA˙^+^ intermediate (lifetime of 200 µs). Subsequently, an electrochemistry-neutral reionization-mass spectrometry (EC-NR-MS, Fig. 3e) technique was developed for the identification of the transient neutral radicals TPrA˙.^49^ The team placed an ion deflector between the spray port and the MS inlet to remove TPrA˙^+^, while the passing TPrA˙ will be ionized by electrospray and detected by MS. The detected TPrA˙ helps to clarify the previously unproven ECL reaction mechanism of the [Ru(bpy)_3_]^2+^/TPrA system. Later, the identification of other neutral co-reactant radicals in the electrooxidation of DBAE, TEOA, BIDE, and BIS-TRIS expanded the understanding of the ECL mechanism in various [Ru(bpy)_3_]^2+^/co-reactant couples.

To systematically compare the diverse techniques discussed in this perspective, we have compiled a summary in Table 1, which illustrates the capabilities of each technique in accessing critical mechanistic parameters. This serves as a practical guide for researchers to select the optimal tool for mechanistic optimizations, ultimately fostering advancements like lower detection limits or minimizing interferences.

**Table 1:** Comparison of techniques in ECL mechanism research^a^

Parameter technique	Radical lifetime	Diffusion length	Emitting-layer thickness	Intermediate identity	Reaction rate constants	Spatial emission profiles
ECLM	✓	✓	✓	—	◆	✓
ECL-SECM	✓	✓	—	—	◆	◆
ECLIS	◆	✓	✓	—	◆	◆
EC-MS	—	—	—	✓	—	—
ECL-simulation	◆	◆	◆	—	◆	◆

a ✓ Available, — unavailable, ◆ indirectly accessed.

In another example of this use of EC-MS, the luminol/hydrogen peroxide (H_2_O_2_) system was investigated. The goal was to provide direct molecular evidence of the proposed intermediates or validate their roles in the ECL processes at the molecular level. But this remains a challenging task. Min *et al.* developed a real-time ECL-MS platform by integrating a photomultiplier tube with EC-MS to elucidate the ECL reaction mechanism (Fig. 3f).^47^ They verified the activity of the reaction intermediates at different pH from the molecular level. Furthermore, the two ECL pathways of luminol were confirmed by observing the key intermediates α-hydroxy hydroperoxide and diazonium quinone and analyzing their relationship with applied voltage and ECL intensity, thereby confirming the previously speculated reaction pathway (Fig. 3g). Furthermore, EC-MS supported the understanding of the possible mechanism for cathodic ECL of L012 (a luminol analog).^50^

### 2.4 ECL simulation

Finite element analysis (FEA) is a method that uses mathematical approximations to simulate real physical systems including the kinetic and thermodynamic parameters. By designing physical models, defining boundary conditions, and selecting dynamic and thermodynamic parameters, finite element simulation can realize the simulation of complex reaction processes. Thus, finite element simulation has developed as a powerful tool to analyze the kinetic data that are difficult to measure using conventional methods.^51–54^ In addition, the concentration profiles and the distribution of ECL and radicals can also be obtained from simulations.^55^

#### 2.4.1 ECL curve simulation

**Fig. 4:**
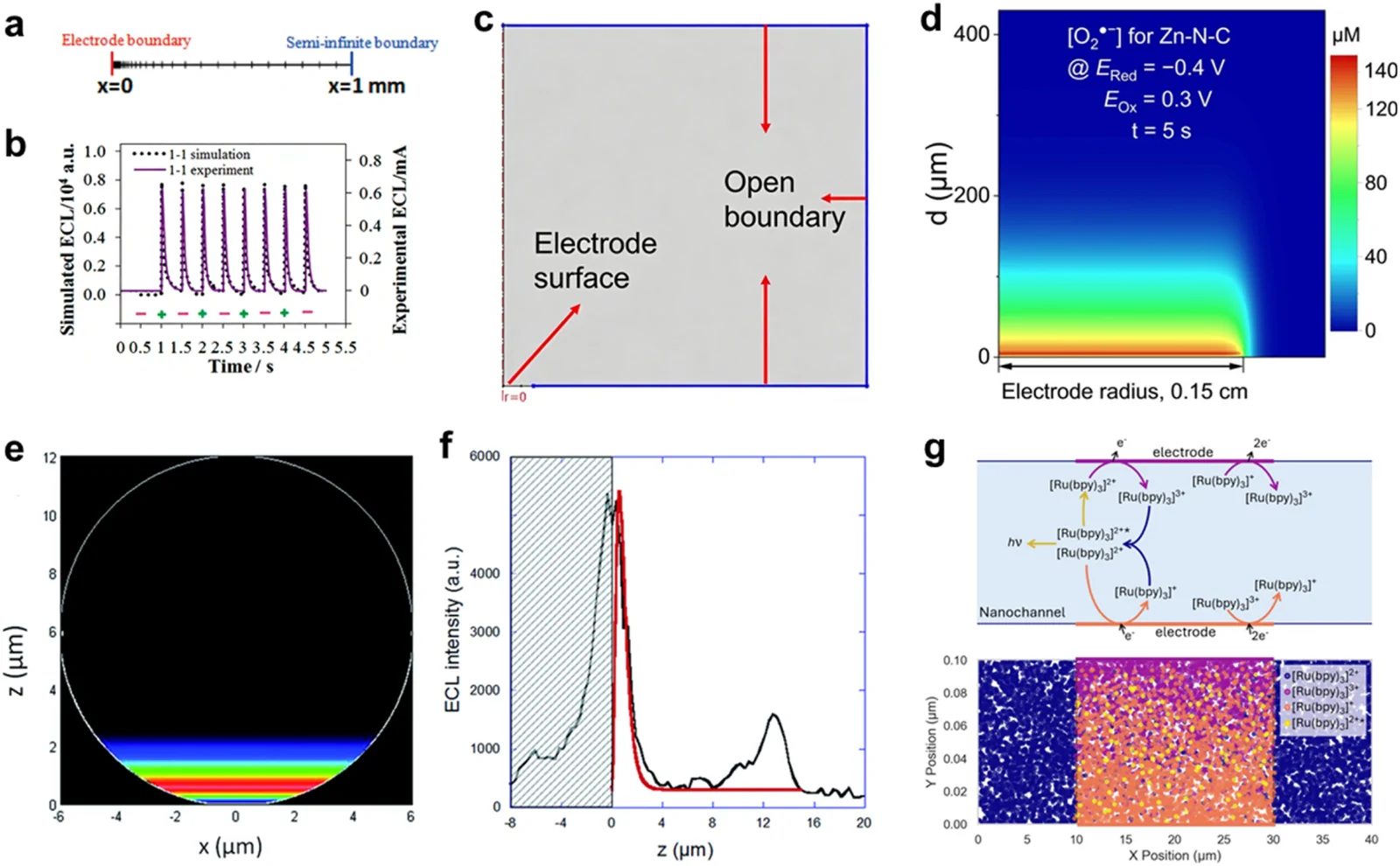
(a) 1D and (c) 2D simulation model in finite element simulations space with spatial simulation elements and the relevant boundaries. Adapted from ref. 57 with permission from the *American Chemical Society*, copyright 2010. (b) The simulation (dotted) and experimental (continuous) transients on the same scale. Simulated side-view image showing the distribution of (d) [O_2_˙^−^] in the diffusion layer of electrocatalyst and (e) [Ru(bpy)_3_]^2+^* on the surface of PS bead. Adapted from ref. 55 with permission from John Wiley and Sons, copyright 2023. (f) Comparison between simulated (red curve) and experimental (black curve) ECL intensity profile of a PS microbead. Adapted from ref. 19 with permission from the *Royal Society of Chemistry*, copyright 2014. (g) Schematic of ECL annihilation reaction in a nanofluidic channel (top) and corresponding stochastic simulation of ions undergoing a random walk as well as reacting in a two-dimensional channel geometry (bottom). Adapted from ref. 20 with permission from the *American Chemical Society*, copyright 2025.

One type of ECL mechanism simulation is based on the simulation of the ECL curve.^56^ Bard and co-workers found that annihilation reactions of different charge combinations (such as radical anion–cation (A˙^−^–A˙^+^) annihilation and anion-dication (A˙^−^–A^2^˙^+^) annihilation) lead to asymmetric ECL transient signals. Digital simulations (one-dimensional (1D) simulations) of transient ECL experiments (Fig. 4a and c) revealed that this asymmetry originates from differences in the amount of generated charge rather than the stability of the species.^57^ In addition, the mechanism of the ECL of luminol involves many reaction processes with different kinetic parameters, but by simulation, the rate constant for the oxidation of luminol by O_2_˙^−^ (6 × 10^5^ M^−1^ s^−1^) can be well determined.^58^ Recently, Chen *et al.* developed a theoretical model with the Langmuir isotherm to describe the adsorption of luminol. Through simulating the concentration profile of 3-aminophthalate ion, the species emitting ECL, they suggested that the ECL intensity could be modulated by the adsorption effect of the luminophore.^59^

Compared to traditional 1D models, 2D models can handle diffusion geometry more realistically.^60^ Su *et al.* demonstrated enhanced ECL using an ITO electrode in a co-reactant system of a 2D model.^61^ The simulated results were in good agreement with experimental observation, indicating that the variation of ECL intensity is mainly associated with the electrochemical oxidation of [Ru(bpy)_3_]^2+^ and TPrA. Zhang's group employed 2D models (Fig. 4b) to adeptly demonstrate their capability in analyzing the reaction kinetics of various reactions related to ECL.^55^ Recently Ding and co-workers presented a novel method to study the charge-transfer rate constants governing all three excited-state generation mechanisms in the [Ru(bpy)_3_]^2+^/tri-*n*-propylamine (TPrA) coreactant system.^62^ Considering that the co-reactant (reactive oxygen species, ROS) for the anodic ECL of luminol is supplied by cathodic oxygen reduction reaction (ORR), they provided an intermediate-oriented method for ORR kinetics analysis. Benefiting from the various ultra-sensitive stoichiometric reactions between ROS and luminol, they obtained relevant quantitative kinetic data, such as the apparent reaction rate constant of ORR and the time/space-dependent variation of ROS (Fig. 4d), by performing FEA on the ECL decay curves. In addition to conventional macroelectrodes, for ultramicroelectrodes, 2D model simulations are also effective for extracting crucial chemical information (*e.g.*, reaction mechanisms and intermediate species) and kinetic data (*e.g.*, rate constants, reaction kinetics, and mass transport kinetics) from ECL systems.^60^

#### 2.4.2 ECL imaging simulation

More recently, ECL imaging was also investigated and simulated. The primary role of ECL imaging simulation is to validate the experimental data. By establishing a reaction-diffusion model, simulations can reproduce the spatial distribution and temporal changes of ECL intensity, thereby determining whether the experimental results conform to basic physicochemical laws. For example, in single-particle or microsphere systems, simulations can be used to reproduce the morphology of the luminescent region near the electrode and compare it with experimental images.^54,63^ Simulations that are consistent with experimental data indicate that the set reaction pathways and kinetic parameters are reasonable.

Furthermore, simulations are often used to verify the hypothetical models proposed by researchers. In ECL imaging, experimental phenomena can often be explained by multiple mechanisms, such as intermediate diffusion control, reaction rate limitation, or interface structure effects. By parameterizing different conditions in the simulation, it is possible to observe whether the calculated results can reproduce the experimental phenomena, thereby screening for the most reasonable mechanism. For example, in classic [Ru(bpy)_3_]^2+^/co-reactant systems, the thickness of the ECL emission layer is closely related to the lifetime of the intermediates. By simulating the emission distribution under different radical lifetime conditions, the hypothesis that the emission region is determined by the diffusion of short-lived intermediates can be verified.^40^

In addition to its role in validation and support, some studies use ECL simulation to reveal the reaction mechanism itself. Simulation is not only used to fit results but also to extract key kinetic parameters or analyze reaction pathways. By numerically simulating the concentration gradient distribution of [Ru(bpy)_3_]^2+^* (Fig. 4e), the characteristic profile of the experimentally observed ECL intensity has been verified.^19^ As the ECL profile is constrained by the concentration gradients of TPrA radicals at the bead surface, thus the key kinetic parameter, the TPrA deprotonation rate constant of 2920 s^−1^, has been determined (Fig. 4f). In addition, the simulation revealed the spatial confinement of the ECL emission region; in the TPrA system, the ECL signal mainly emanates within 3 µm of the electrode surface.^19^ In another work, simulations showed that in confined spaces, the local accumulation of intermediate concentrations can significantly alter the reaction pathway, thereby affecting the luminescence efficiency.^64^ These results theoretically explain the nature of nanoconfinement-enhanced ECL and provide important guidance for designing high-performance systems.

It is worth noting that there are also cases where the fitting outcomes in ECL simulations differ from the experimental images. This is usually caused by too many assumptions, inaccurate kinetic parameters, and other factors that make the simplified model deviate too much from the actual model.^36,41^ Furthermore, traditional ECL simulation relies on the finite element method, but in mesoscopic scales or environments with a small number of particles (such as nanochannels), the continuum assumption fails, and stochastic effects need to be considered. Recently, Fracassa *et al.* combined kinetic constants derived from density functional theory (DFT) calculations and finite element simulation to disentangle the radical decomposition rates from experimental artifacts such as concentration, pH, and buffer effects. This approach combining experimental and computation chemistry explicitly establishes the concept of an optimal radical half-life window for bead-based ECL systems. Within this range, radicals are sufficiently long-lived to reach relatively distant luminophores yet remain reactive enough to sustain ECL generation.^65^

Mathwig proposed generating Python scripts through ChatGPT to build an ECL reaction simulator based on a 2D random walk algorithm,^20^ aimed at studying ECL reactions in nanofluidic channels (Fig. 4g).^66,67^ It should be noted that the application of AI tools needs to be combined with solid knowledge of chemistry and physics, and strict validation is required to ensure scientific accuracy.

## 3 Emerging mechanistic strategies for ECL enhancement

From the nature of the reaction pathway, the generation of ECL depends on four consecutive processes: electron transfer on the electrode surface, formation and diffusion of reaction intermediates, generation of excited-state species, and photon emission. Improving the efficiency or reducing the loss of any one of these processes can enhance the ECL signal. The core mechanisms of ECL enhancement can be summarized as three fundamental strategies: first, accelerating the efficiency of electron transfer and reducing the reaction energy barrier; second, increasing the yield of excited state species and reducing non-radiative transitions; third, optimizing the reaction microenvironment to maintain sustained and efficient reactions. Based on the above enhancement concepts, various approaches have been developed, such as catalytic enhancement by co-reactants, electron transfer enhancement, confinement and spatial enrichment enhancement, aggregation induced emission (AIE)/aggregation-state enhancement, self-enhanced, surface plasmon resonance (SPR), resonance energy transfer (RET), electromagnetic field enhancement, microfluidics and kinetic enhancement, and so on. In the past five years, some interesting approaches have emerged, involving the principles of the peroxydisulfate-oxalate mechanism, redox mediators, temporal regulation and exogenous stimulus.

### 3.1 Peroxydisulfate-oxalate mechanism

Conventional ECL systems are generally described through two main routes, the oxidative–reduction and reductive–oxidation pathways.^68^ In the benchmark [Ru(bpy)_3_]^2+^/TPrA oxidative–reduction co-reactant system, ECL is typically triggered only at relatively high anodic potentials, around 1.4 V *vs.* Ag/AgCl, mainly due to the sluggish co-reactant oxidation.^69^

**Fig. 5:**
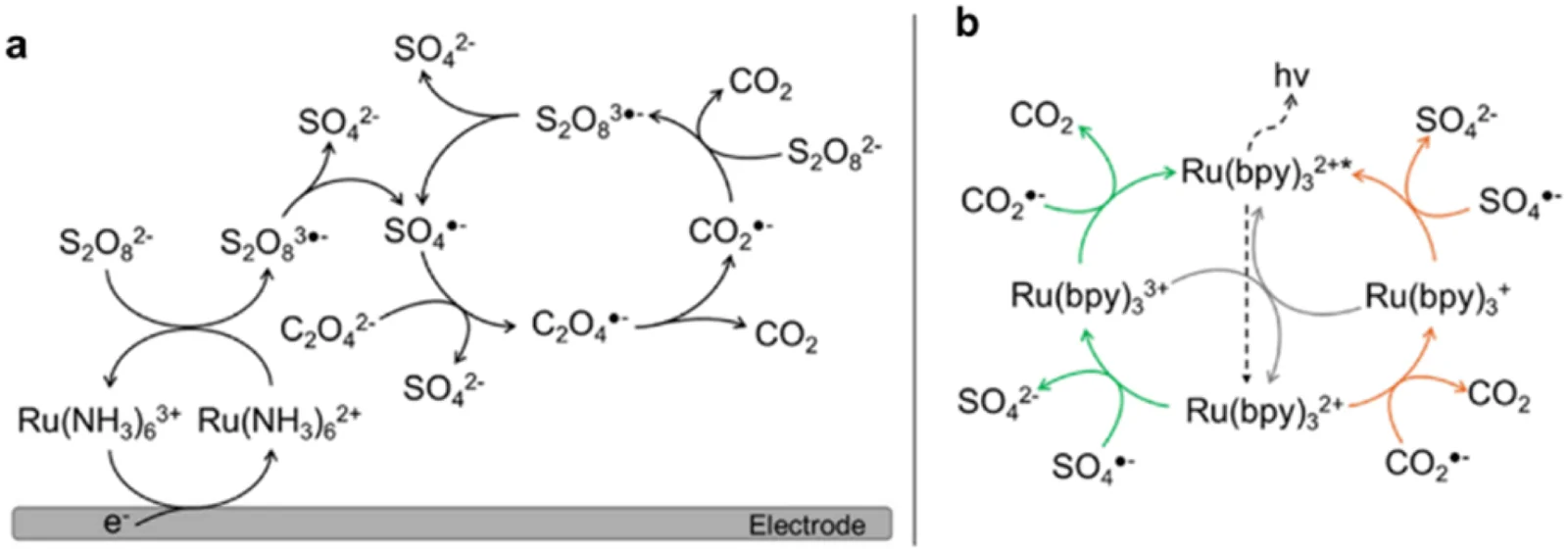
Schematics of (a) the electrochemical peroxydisulfate-oxalate reaction on a GC working electrode and (b) the reductive excitation (green arrows) and oxidative excitation (orange arrows) pathways to generate ECL from the [Ru(bpy)_3_]^2+^ luminophore after the catalytic cycle is closed as described in (a). Adapted from ref. 23 with permission from the John Wiley and Sons, copyright 2025.

These operating potentials can promote electrode-surface modification,^70^ parasitic processes such as oxygen evolution, and increased background noise, all of which compromise performance in sensitive bioanalysis and imaging.^22,23,71^ White and co-workers recently reported a new strategy to generate highly energetic SO_4_˙^−^ and CO_2_˙^−^ radicals, from peroxydisulfate (S_2_O_8_^2−^) and oxalate (C_2_O_4_^2−^) ions, respectively, initiated by the outer-sphere redox mediator [Ru(NH_3_)_6_]^3+^ under remarkably mild electrochemical conditions (*i.e.*, −0.2 V *vs.* Ag/AgCl) (Fig. 5a).^22,72^ Along this line, Valenti *et al.* introduced an peroxydisulfate-oxalate co-reactant ECL pathway that offers a fundamentally different approach by lowering the triggering potential to about −0.2 V *vs.* Ag/AgCl, avoiding direct oxidation of the luminophore at the electrode surface (Fig. 5b).^23^ In this mechanism, electrochemical reduction of [Ru(NH_3_)_6_]^3+^ produces [Ru(NH_3_)_6_]^2+^, which transfers a single electron to S_2_O_8_^2−^ to generate SO_4_˙^−^. The sulfate radical then oxidizes oxalate to produce CO_2_˙^−^, and this reducing radical in turn reacts with fresh S_2_O_8_^2−^ to regenerate SO_4_˙^−^, thereby sustaining a catalytic cycle. The result is the simultaneous production of strongly oxidizing and strongly reducing radicals in solution at very low applied potential.

The key consequence of this cascade of reactions is that [Ru(bpy)_3_]^2+^ can be excited through homogeneous radical reactions without undergoing direct oxidation or reduction at the electrode. This low-potential mechanism also generates a thin emitting layer (around 5 µm) resulting in a stable signal. Moreover, this novel ECL excitation process can be extended to more energy-demanding luminophores, including blue-emitting Ir(iii) complexes with large band gaps that are inaccessible through the traditional ‘remote’ TPrA route. Thus, the peroxydisulfate-oxalate mechanism represents more than a new co-reactant system: it introduces a new mechanistic framework for ECL enhancement based on reaction-network design, offering lower electrochemical stress, reduced parasitic chemistry, and promising opportunities for safer and more efficient bioanalysis and ECL microscopy.

### 3.2 Redox mediated mechanism

**Fig. 6:**
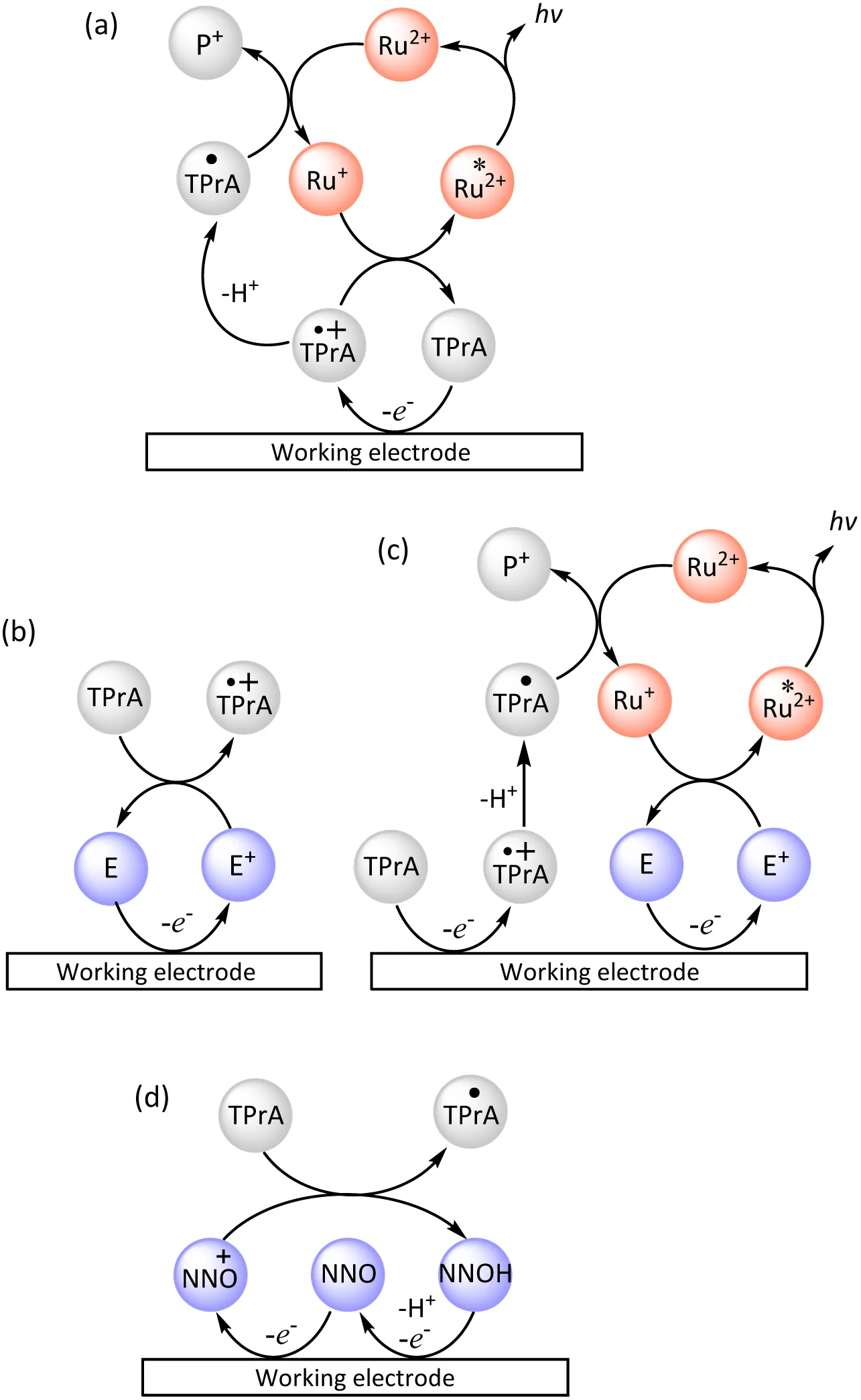
(a) The conventional oxidative (TPrA˙^+^) excitation pathway of [Ru(bpy)_3_]^2+^ (Ru^2+^) with TPrA as a co-reactant, also known as the ‘indirect’ or ‘remote’ route. (b) Electrocatalysis of TPrA by a redox mediator, E. (c) An alternative oxidative (E^+^) excitation pathway. (d) Electrocatalysis and co-reaction acceleration by nortropine *N*-oxyl (NNO).

Most analytical applications of ECL involve electrochemical oxidation or reduction of co-reactants that generate reactive radical intermediates capable of exciting the luminophore.^73^ Although these pathways can be highly effective, the ECL intensity is fundamentally limited by the rates at which these co-reactant intermediates are generated, and their ability to diffuse to the luminophore. These aspects are well illustrated by the commercial application of microbead-supported assays with ECL detection for clinical diagnostics.^74^ Within these systems, the biomolecule-bound Ru(ii)-complex luminophore is too far from the electrode for direct electron transfer, and its excitation therefore depends on the diffusion of co-reactant intermediates from the electrode surface to the luminophore (Fig. 6a). The TPrA˙^+^ radical cation has a lifetime of ∼0.2 ms, sufficient to diffuse to a portion of the immobilized luminophores, but its electrogeneration from the TPrA co-reactant is relatively slow unless high overpotentials are applied.^39,74^

**Fig. 7:**
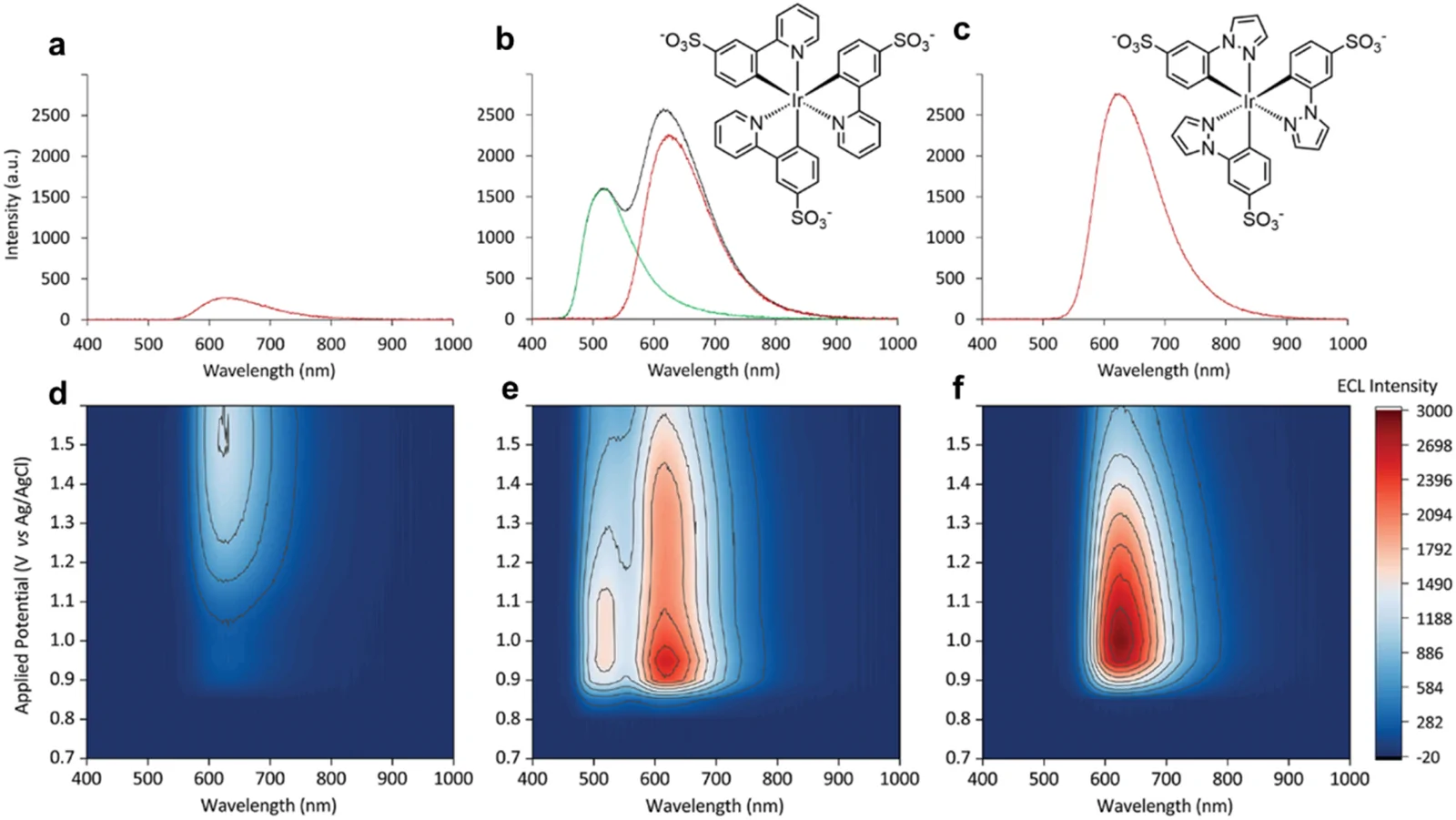
(a–c) ECL spectra of 1 µM [Ru(bpy)_3_]^2+^ in ProCell buffer containing 180 mM TPrA, with (a) no redox mediator, (b) 100 µM [Ir(sppy)_3_]^3−^, or (c) 200 µM [Ir(sppz)_3_]^3−^, when applying a potential of 0.95 V *vs.* Ag/AgCl. In (b), the spectrum (black plot) has been deconvoluted into the contributions from [Ru(bpy)_3_]^2+^ (red plot) and [Ir(sppy)_3_]^3−^ (green plot). (d–f) ‘3D ECL’ contour plots of the same reaction mixtures, showing the ECL spectral distribution over a series of applied potentials (50 mV intervals). Adapted from ref. 43 with permission from the John Wiley and Sons, copyright 2024.

One approach to overcome these limitations is the introduction of ‘redox mediators’: molecules or ions that can be reversibly oxidized and reduced, facilitating electron transfer between an electrode and redox-active species in solution.^24,75^ Redox mediators have been widely applied in areas such as electrocatalysis, electrosynthesis, and energy storage, providing alternative electron-transfer pathways that improve electrochemical efficiency and control over reactivity.^76^ In the context of ECL, redox mediators have been shown to (i) catalyze co-reactant oxidation (Fig. 6b), and (ii) provide an alternative, more stable intermediate for the oxidative excitation^77^ of the luminophore (Fig. 6c).^10,43,53,78,79^ In this second role, the mediator participates directly in the excitation chemistry rather than solely shuttling electrons. In combination, these additional pathways not only increase the ECL intensity, particularly at low potentials (Fig. 7), but also enable excitation of luminophores further away from the electrode surface.^43^

The redox-mediator approach to enhance ECL conceptually complements that of ‘co-reaction accelerators’, which catalyze the dissociation of co-reactants into their radical intermediates.

To date, however, research into accelerators has been predominantly focused on nanomaterial catalysts for reductive-oxidation co-reactant systems (*e.g.*, H_2_O_2_, O_2_, S_2_O_8_^2−^), often using luminol as a precursor to the emitting species.^80^ In contrast, small-molecule redox mediators have been investigated primarily to increase the ECL of [Ru(bpy)_3_]^2+^ and related metal complexes with the oxidative–reduction co-reactant TPrA and related tertiary amines.

The defining attributes of effective redox mediators for ECL have been established using Ir(iii) complexes bearing peripheral polar groups (sulfonate or tetraethylene glycol) that provide high solubility in aqueous solution, in conjunction with [Ru(bpy)_3_]^2+^ as the primary luminophore.^21,24,81^ Favorable properties include: (i) chemically and electrochemically reversible oxidation at a potential close to that of the irreversible oxidation of the co-reactant; (ii) not reducible by TPrA˙; and (iii) greater excited-state energy than the luminophore to avoid quenching *via* energy transfer. To date, the mediator providing the greatest increase in the co-reactant ECL of [Ru(bpy)_3_]^2+^ is [Ir(sppy)_3_]^3−^ (Fig. 7b).^24^ With the [Ru(bpy)_3_]^2+^ luminophore free in solution, the addition of 100 µM [Ir(sppy)_3_]^3−^ elicited an 11-fold increase in the ECL intensity at an applied potential of 0.9 V *vs.* Ag/AgCl, but only 1.5-fold at 1.2 V. When applied to [Ru(bpy)_3_]^2+^ immobilized on 12 µm polystyrene beads (mimicking the detection conditions of microbead-supported sandwich immunoassays), the ECL enhancement was 71-fold and 2.9-fold at the same two potentials.^78^

**Fig. 8:**
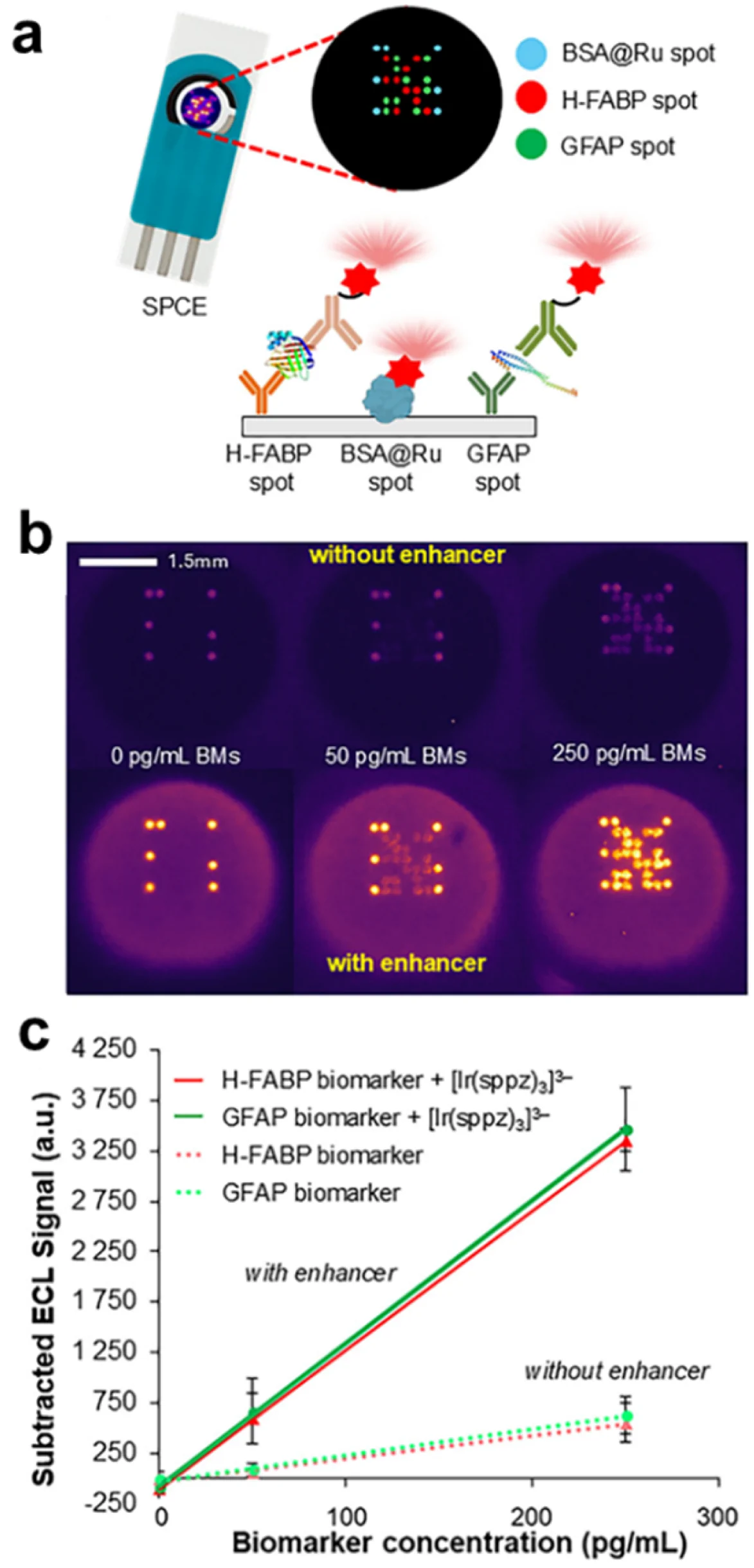
(a) A spatially resolved ECL immunoassay for two biomarkers: (heart-type fatty acid-binding protein (H-FABP) and glial fibrillary acidic protein (GFAP)), using a microarray patterned on a screen-printed electrode. (b) Representative ECL images obtained in ProCell buffer (containing 180 mM TPrA) without and with the [Ir(sppz)_3_]^3−^ redox mediator (100 µM) at 1.25 V *vs.* Ag. (c) Background-subtracted ECL intensities as a function of biomarker concentration. Control spots contained 0.8 pg of Ru(ii)-complex-labelled BSA per spot. Error bars represent ±1*σ* across 10 spots from three independent electrodes. Adapted from ref. 85 with permission from the *American Chemical Society*, copyright 2026.

The [Ir(sppy)_3_]^3−^ mediator, however, simultaneously emits green ECL through the reductive excitation (direct) co-reactant pathway with TPrA (Fig. 7b and e). Distinguishing the ECL of the primary luminophore from that of the mediator, *via* spectral deconvolution,^24^ background subtraction,^78^ and/or optical filtration,^10^ somewhat offsets the improvement in analytical sensitivity. The sulfonated tris(1-phenylpyrazolato)iridium(iii) analogue ([Ir(sppz)_3_]^3−^; Fig. 7c) was therefore developed as a non-emissive alternative.^43^ With the [Ru(bpy)_3_]^2+^ luminophore in solution, 200 µM [Ir(sppz)_3_]^3−^ gave an 11-fold increase in ECL intensity at 1.0 V, and 4-fold at 1.3 V *vs.* Ag/AgCl. When the luminophore was immobilized on 1 µm beads, the enhancement was 6.2-fold and 2.3-fold, at the same two potentials.^43^ This redox mediator was utilized for a 5-fold increase in analytical sensitivity for two biomarkers (in a spatially resolved ECL immunoassay on screen-printed electrodes (Fig. 8)).^10^ Under these conditions, where the capture antibody is bound to the electrode surface, the reductive excitation ECL pathway may also contribute to the observed emission, *via* multistep electron-transfer through the protein.^82–84^ For the application to biological samples, however, challenges remain in the redox-mediator enhancement of interfering emissions,^85^ both from non-specific binding of the detection antibody, and the inherent ECL background of the TPrA co-reactant ascribed to the formation of singlet dioxygen.^86^

The sulfonated redox mediators and their parent neutral complexes were recently examined as enhancers of Ir(iii) luminophores with TPrA in aqueous and acetonitrile solutions, respectively.^87,88^ In some systems, such as [Ir(sppy)_2_(bpy)]^−^ with the [Ir(sppy)_3_]^3−^ mediator, the degree of enhancement was similar to that of [Ru(bpy)_3_]^2+^. In other cases, the redox mediator was not effective, because the required excitation pathways were either thermodynamically inaccessible or less efficient than competing routes.^87,88^

The Ir(iii)-complex-based redox mediators effectively promote co-reactant ECL under a variety of conditions, but more cost-effective alternatives such as organic compounds would be beneficial. A carbazolyl dicyanobenzene compound used to increase the ECL of an Ir(iii) luminophore bis(2-phenylbenzothiazolato)(acetylacetonate)iridium(iii) (Ir(bt)_2_(acac)) in a light-emitting device was referred to as a redox mediator.^89^ Like the pathway shown in Fig. 6c, the organic compound participated directly in the excitation of the luminophore, but in this case,^89^ the mechanism was akin to ‘mixed’ annihilation ECL involving two different precursor molecules in solution,^90–92^ which is distinct from conventional co-reactant ECL.

**Fig. 9:**
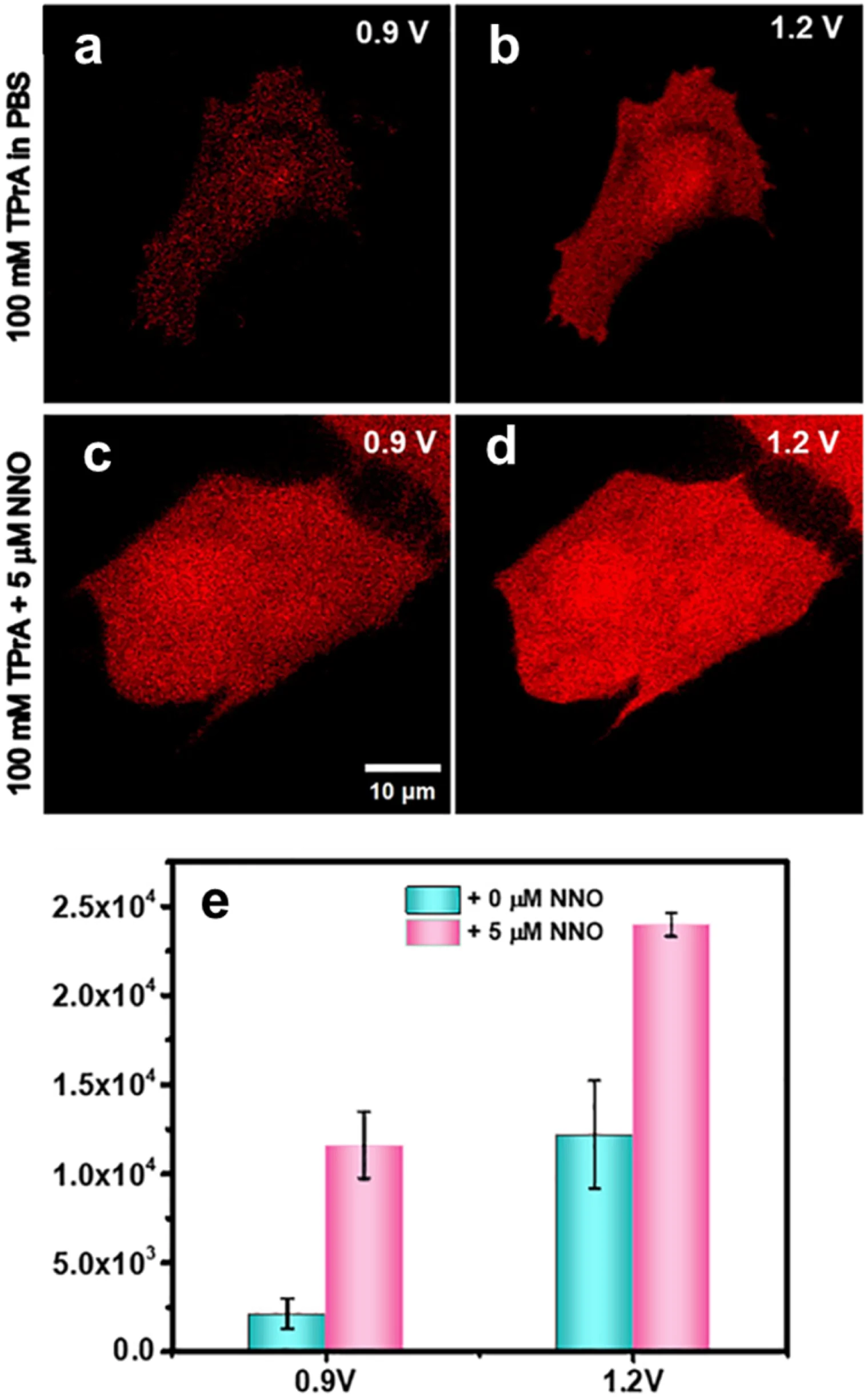
(a–d) ECL images of [Ru(bpy)_3_]^2+^ labelled cells in 100 mM PBS (pH = 7.5) containing (a and b) 100 mM TPrA, and (c and d) 100 mM TPrA and 5 µM NNO, at 0.9 V and 1.2 V *vs.* Ag/AgCl. (e) Relative ECL intensities of the labelled cells at the two applied potentials (*n* = 4). Adapted from ref. 93 with permission from the John Wiley and Sons, copyright 2025.

Nortropine *N*-oxyl (NNO) was recently reported as a non-emissive, organic redox mediator for the co-reactant ECL of [Ru(bpy)_3_]^2+^ and TPrA (or DBAE).^93^ Interrogation of the mechanism revealed that the electrochemically oxidized mediator could provide an alternative to TPrA˙^+^ for the oxidative excitation of the luminophore (Fig. 6c), or accept a hydrogen atom from TPrA to form TPrA˙, effectively combining electrocatalysis and co-reaction acceleration (Fig. 6d). The addition of 5 µM NNO decreased the ECL intensity of [Ru(bpy)_3_]^2+^ with TPrA in homogeneous solution, but amplified the ECL 7.3-fold at 0.8 V and 1.9-fold at 1.2 V *vs.* Ag/AgCl when the luminophore was immobilized on 12 µm beads, without significant enhancement of the background emission.^93^ The NNO mediator was applied to the ECL microscopy of single cells. Chinese hamster ovary cells were cultured on the working electrode surface and labelled with a streptavidin-modified [Ru(bpy)_3_]^2+^ derivative, *via* biotinylated proteins present on the cellular membrane. Addition of 5 µM NNO increased the ECL of the label by 5.4-fold at 0.9 V and 2-fold at 1.2 V *vs.* Ag/AgCl (Fig. 9). Based on these promising findings, there is clearly considerable scope for the development of novel metal-complex and organic redox mediators for substantial enhancement of co-reactant ECL systems.

### 3.3 Time-scale regulation

In the early stages of research on enhanced ECL, distance regulation emerged as a core method. For example, by adjusting the distance between the electrode and the luminophore, the electron transfer efficiency can be optimized;^94–96^ similarly, by adjusting the distance between the luminophore and the reactive molecules, the probability of radical collisions can be increased,^97,98^ both of which greatly enhance the ECL. In principle, the physical and chemical properties of materials are intrinsically bestowed by interplays not only over different length scales but also at variable time scales.^12^ From the ECL reaction pathways described above, it is evident that prolonging the lifetime of the electrogenerated radicals is an important approach to enhance ECL.^99–101^

#### 3.3.1 Radical stabilization

Extending the lifetimes of the excited state and co-reactant radicals can increase the probability of radiative recombination and enhance ECL efficiency, breaking through the previous limitation where distance control could only reduce quenching but could not extend the lifetime of the excited state and radicals.^102^ Considering the key reaction between cation and anion radicals during ECL emission, several groups claimed that the radical stabilization may promote excited state generation. For example, taking advantages of D–A pairs, a luminescent covalent organic framework (COF) was designed as an ECL emitter by integrating triazine and triphenylamine as donor and acceptor units in the reticular structure, respectively.^97^ Due to the stable radical triazine anions and rapid intrareticular charge transfer (IRCT), highly crystalline triazine based COF showed about 123-fold enhancement of ECL compared to the benzene-based COF.

**Fig. 10:**
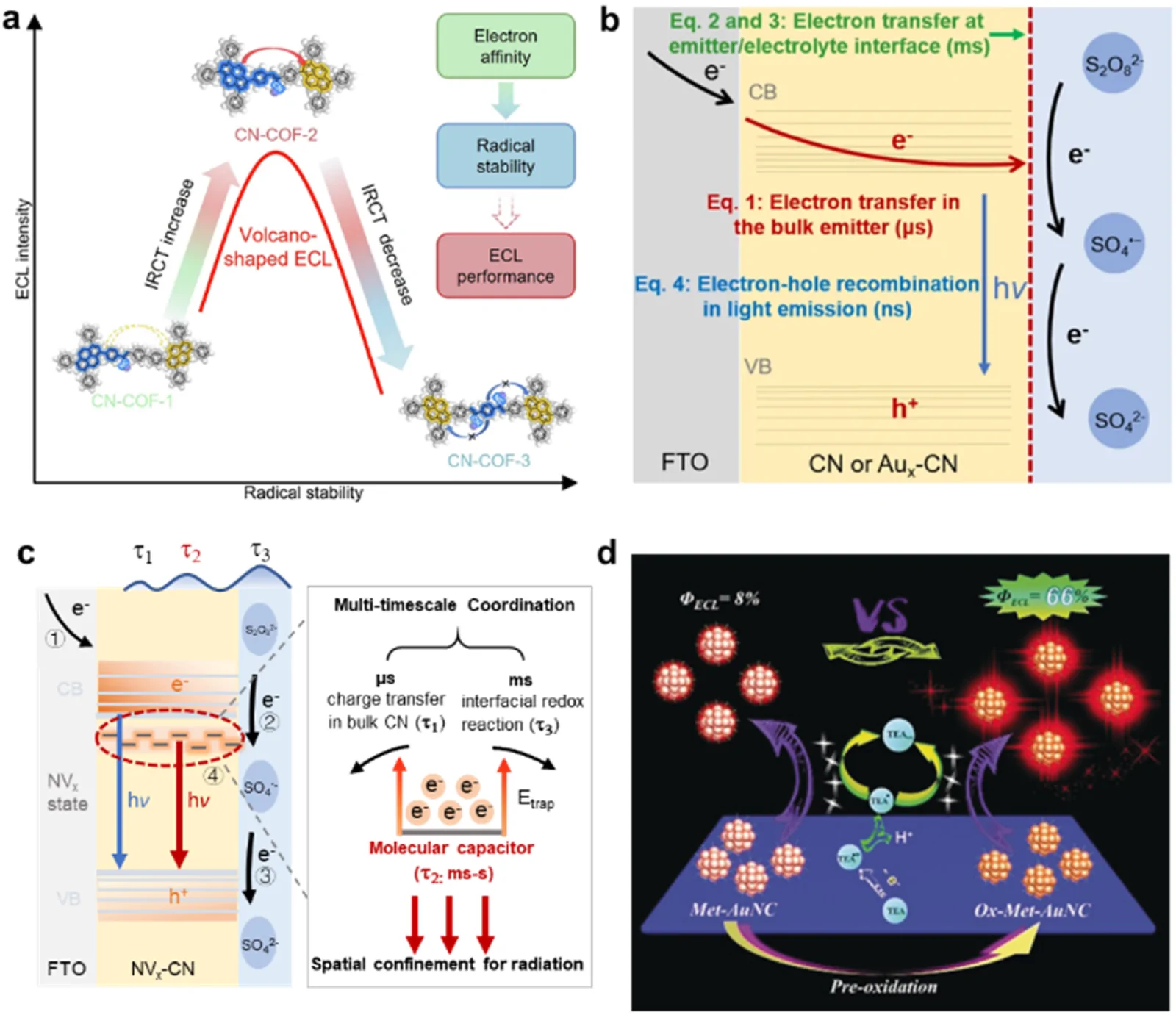
(a) Dependence of volcano-shaped ECL on radical stability of CN-COFs *via* IRCT between pyrene units. Adapted from ref. 98 with permission from the Springer Nature, copyright 2025. Possible charge transfer processes of (b) shallow trap states ECL and (c) molecular capacitors ECL. Adapted from ref. 11 with permission from the Springer Nature, copyright 2024. Adapted from ref. 104 with permission from the John Wiley and Sons, copyright 2025. The ECL processes of ‘1–4’ in (c) represent electron injection, bulk electron transport, interface electron transfer, and radiative recombination, respectively. (d) Schematic of enhanced ECL performance after the pre-oxidation treatment. Adapted from ref. 105 with permission from the John Wiley and Sons, copyright 2019.

Furthermore, the anion radicals generated during ECL could be stabilized by the electron-withdrawing acrylonitrile linkers, providing a radical stability-dependent ECL generation. Three acrylonitrile linked covalent organic frameworks (CN-COFs) by cyanomethyl contained monomers were synthesized, and the relationship between the radical stability of these materials and their ECL properties were investigated.^98^ Because CN–COF-1 has weaker electron affinity, the anion radicals it produces are in an ‘unstable’ state, resulting in lower ECL intensity. Later, by increasing the stability of the radicals and enhancing the charge transfer efficiency between COF˙^−^ and COF˙^+^, the intensity and duration of ECL were improved. However, when the electron affinity of CN–COF-3 is further increased, the overly stabilized COF˙^−^ hinders the transfer of charge to COF˙^+^, leading to a reduction in ECL performance (Fig. 10a).

#### 3.3.2 Time matching

Typical ECL with co-reactants involves multiple charge-transfer pathways, including electron transfer in bulk emitters, redox reactions at emitter/co-reactant interfaces, and electron transition between excited and ground states.^103^ Notably, however, there are huge timescale mismatches among these processes, from picoseconds to seconds. Thus, reconciling their timescale could be anticipated to open a new methodology to further boost ECL.^11,104^ Focusing on the time-scale correlation effects of shallow trap states revealed that the time gap between fast electron transfer and slow surface layer reactions in carbon nitride is a limiting factor for efficiency (Fig. 10b).^11^ By introducing shallow electron traps through Au–N bonds as an electron buffer pool, the multi-time-scale processes from nanoseconds to seconds are bridged, achieving precise matching of charge transfer kinetics, enhancing the efficiency of excited-state generation and radiative decay, and establishing a full-time-domain quantitative kinetic characterization method.

Another time-matching approach involves the construction of molecular capacitors to facilitate spatiotemporal electron cooperative enhancement of carbon nitride ECL. This technique addresses the disparity between electron transfer kinetics and interfacial redox timescales, along with the spatial competition of electrons between radiative and non-radiative pathways (Fig. 10c).^104^ By leveraging nitrogen vacancies and cyano terminal groups to establish electron capture and accumulation sites, electrons are spatially confined. Simultaneously, the relaxation process harmonizes rapid charge transfer with slower interfacial reactions. This significantly reduces electron loss and amplifies effective radiative recombination, ultimately boosting ECL efficiency by almost two orders of magnitude, providing a novel mechanism for highly sensitive sensing.

#### 3.3.3 Pre-oxidation/reduction

Pre-oxidation and pre-reduction refer to processes implemented before the main ECL reaction to regulate the concentration and lifetime of active intermediates (such as free radicals and excited-state precursors) in advance. For example, Liu and co-workers^105^ controlled the pre-oxidation time of l-methionine-stabilized gold nanoclusters. At 15–25 s, the oxidation of the ligands on the nanocluster surface exhibited the highest electron transfer efficiency, and the ECL quantum yield reached 66%, significantly enhancing the anodic ECL emission intensity (Fig. 10d). Lei *et al.* accumulated stable cation radicals by regulating the pre-reduction time of electroactive metal–organic frameworks (E-MOF), ultimately enhancing the experimental anodic ECL signal.^106^ The self-enhanced ECL mechanism of E-MOF was proposed that S_2_O_8_^2−^ could be pre-reduced to produce SO_4_˙^−^, then reacted with E-MOF, leading to E-MOF˙^+^. Once the E-MOF was further reduced to E-MOF˙^−^, it could annihilate with the accumulated E-MOF˙^+^ to produce E-MOF* for strong ECL emission, providing a new platform for self-enhanced ECL. Notably, excessively prolonged pre-oxidation time would lead to nanocluster structure damage and decreased luminescence efficiency.

### 3.4 Exogenous stimuli

Exogenous stimulus enhancement of ECL differs from material modification and device upgrading. It achieves significant improvements in luminescence intensity, spatiotemporal resolution, and imaging contrast by introducing external field energy to break the limitations of reaction kinetics. The three physical external fields of light, heat, and ultrasound have advantages such as non-contact, localization, and simple temporal control, and have become important directions in fundamental ECL research and imaging applications.

#### 3.4.1 Heat

**Fig. 11:**
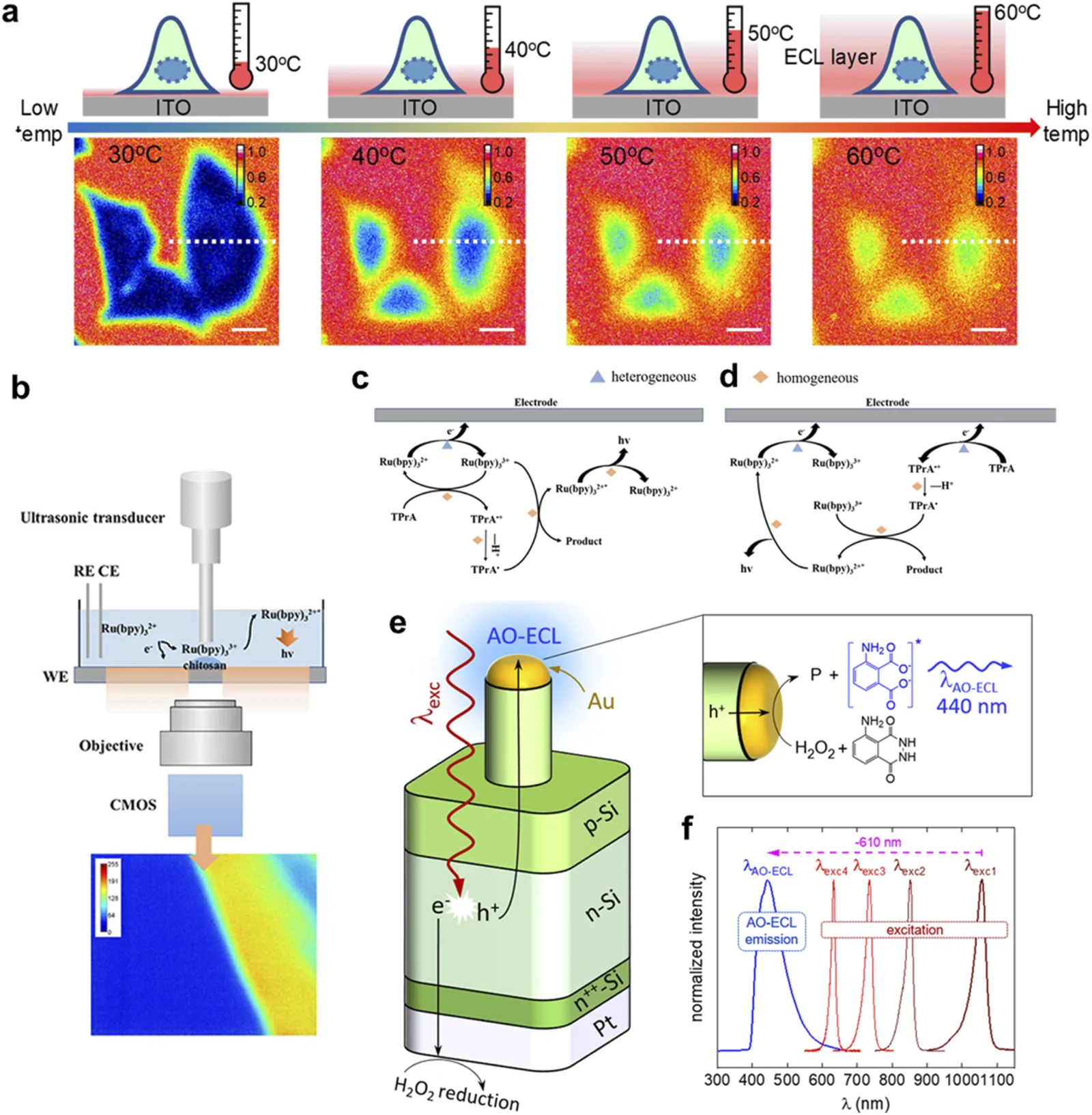
(a) Schematic illustration of ECL layer evolution with elevated temperature from 30 °C to 60 °C. The electrode surface is modified with an attached cell. The ECL layer gradually thickens with higher temperatures (top); shadow ECL patterns of three cells at different temperatures from 30 °C to 60 °C (bottom). Adapted from ref. 111 with permission from the Springer Nature, copyright 2022. (b) Schematic of the ultrasound-ECL apparatus used to measure the patterns and the corresponding ECL images. Schemes for the mechanisms involved in the [Ru(bpy)_3_]^2+^/TPrA coreactant ECL system: (c) catalytic route and (d) oxidative–reduction route. Adapted from ref. 114 with permission from the *American Chemical Society*, copyright 2023. (e) Scheme of monolithic AO-ECL at a nanostructured Au-*pnn*^++^Si–Pt junction under illumination. Hole transfer mechanism leading to visible photons generation at the solid–liquid interface, P represents reaction products. (f) Normalized spectra of the incident excitation light-emitting diodes (brown/red curves) and the resulting AO-ECL emission of luminol/H_2_O_2_ at Au-*pnn*^++^Si–Pt (blue curve); the largest anti-Stokes shift is represented by a pink dashed arrow. Adapted from ref. 125 with permission from the *American Chemical Society*, copyright 2023.

As a controllable exogenous stimulus, heat offers an effective regulatory pathway for enhancing ECL.^107–110^ The primary mechanism involves optimizing the reaction microenvironment and accelerating reaction kinetics. The reaction layer thickness increases with temperature, especially when the ECL reactions were dominated by the catalytic route.^111^ By adjusting the electrode temperature, the state of the reaction layer can be precisely controlled, allowing for clear imaging of different height morphologies of individual cells (Fig. 11a). On this basis, a laser-mediated selective heating method was developed.^112^ Through the photothermal effect, local high-temperature regions are formed on the electrode surface, which not only increases the ECL intensity at the heating point by 63 times and raises the reaction onset potential by 0.2 V, but also thickens the reaction layer. This selective heating strategy has been successfully applied to imaging of suspended cells, enhancing image contrast approximately 20-fold and broadening the application scope of thermal stimulation in ECL imaging.

#### 3.4.2 Ultrasound

As a non-destructive and biologically friendly process, ultrasound can facilitate the *in situ* formation of co-reactants and acts as a stirring pump that accelerates mass transfer and electrochemical reaction rates, leading to a more intense emission of photons. Several research groups have demonstrated the enhancing effect of ECL@sonication using [Ru(bpy)_3_]^2+^ as an ECL emitter in the presence of different co-reactants including TPrA, oxalate ion, ascorbic acid, and dehydroascorbic acid.^113^ Recently, Ma *et al.* studied the ultrasound effects on ECL intensity under different ECL routes (Fig. 11b).^114^ Observations made using ECL microscopy with an ultrasonic probe demonstrated that within the catalytic reaction pathway (Fig. 11c), ultrasound enhances the ECL intensity. Conversely, in the oxidative–reduction pathway (Fig. 11d), an opposite trend was noted. Simulation results suggest that ultrasound facilitates the direct electrochemical oxidation of TPrA radicals by the electrode, which made the TEL thinner than that in the catalytic route under the same ultrasound conditions.

Ultrasound can split water for *in situ* ROS generation. Zhu *et al.* developed an ECL imaging system under the synergetic effect of ultrasound and electric irradiation.^115^ This not only increases the concentration of dissolved oxygen but also facilitates the *in situ* generation of OH˙, O_2_˙^−^, and other reactive oxygen species. Prolonging the ultrasound treatment time gradually enhances the ECL intensity. This system was utilized for highly sensitive detection of dopamine.

#### 3.4.3 Light

Light irradiation can induce the formation of hot holes at the electrode surface, which are capable of oxidizing ECL reagents. Su *et al.* used photo-induced hot carriers to enhance luminol ECL in the absence of H_2_O_2_.^116^ Significantly, the photo-assisted ECL technique, which utilizes semiconductors as the electrode material and combines photoinduced charge carriers with ECL reactions, has emerged as a significant area of research.^7,117–124^ To be precise, the core role of light illumination in photo-assisted ECL is to trigger the generation of charge carriers, thereby regulating the electron transfer pathway of ECL reaction. Its mechanism can be divided into two modes. One mode, called photoinduced ECL (PECL), is light-illuminated assisted ECL, wherein the light generates electron–hole pairs. The charge carriers participate in the redox reactions of luminophores or co-reactants through interfacial transfer, which reduces the driving potential of electrodes and minimizes side reactions and background signals. The other mode, called all-optical ECL (AO-ECL), is light-driven ECL, wherein the photovoltage generated by light illumination can directly drive the luminescence reaction without the need for additional external circuit power supply, achieving electricity-free luminescence in a ‘light-in, light-out’ manner.^125–128^ The core of this mode is the precise regulation of carrier separation and interfacial charge transfer by light illumination. A typical example is Au-*pnn*^++^Si–Pt-based AO-ECL system (Fig. 11e and f). In this architecture, photogenerated holes at the illuminated surface oxidize luminol, while photogenerated electrons are consumed at the catalytic interface on the opposite side, thereby ensuring that charge balance is maintained throughout the integrated device. Notably, this structure enables anti-Stokes AO-ECL, where low-energy excitation photons can generate high-energy light signals, thereby achieving optical separation between the excitation and emitted light. This mechanism fully illustrates the synergistic effect between light energy input and electrical energy input.

### 3.5 Alternative approaches

The facilitation of charge transfer between the active radicals of the emitters and the co-reactants is crucial in augmenting the efficiency of ECL. This is primarily due to the increased generation of the ECL excited state. Various strategies, such as leveraging the pore confinement effect,^129^ self-enhanced ECL,^130^ SPR,^131,132^ AIE,^133^ RET,^134^ and co-reactant accelerators^80^ can foster the interaction between emitters and co-reactants. These methods have been extensively utilized to amplify ECL efficiency.^13,135^ Several comprehensive reviews have already addressed these enhancements by such mechanisms and therefore a detailed discussion is not presented here. ECL enhancement can achieve high-sensitivity detection not only by using a single mechanism but also through the synergistic enhancement of multiple principles.^14^

For example, Jie *et al.*^136^ used isoluminol/hydrogen-bonded organic framework (ILu/HOF-14) as the ECL system, utilizing the nanoconfined structure of HOF-14 to achieve enrichment and dispersion of the luminophore ILu, combined with the catalytic effect of nanoenzymes to accelerate the oxidation of co-reactants and promote the generation of reactive free radicals. The synergy of the two significantly increased the ECL emission intensity and stability, providing an efficient luminescent platform for biosensing. They also reported an ECL biosensing system that was constructed using COF@Lu (luminol-embedded sea-urchin-like COF) as the luminophore and Cu_*x*_O nanorods as the electrocatalyst.^137^ The nanoscale confinement effect of the COF provided local enrichment of luminol and enhancement of the anodic ECL signal, while the Cu_*x*_O nanorods catalyzed the co-reactant H_2_O_2_ reduction to generate a large amount of reactive oxygen species. The synergy between the two increased the luminol ECL efficiency by 60 times compared to luminol alone.

Wei *et al.*^138^ synthesized a zirconium-based metal–organic framework (Zr-MOF) ECL emitter using AIE luminogen (AIEgen) 1,1,2,2-tetra(4-carboxylbiphenyl)ethylene (H_4_TCBPE) as a ligand. The MOF framework induces aggregation of AIEgen and enhances its ECL efficiency. At the same time, a titanium dioxide@silver nanoparticles (TiO_2_@Ag NPs) heterostructure is combined as a co-reactant accelerator. Zhu *et al.*^139^ confined AIE-featured ECL luminophores within nanocarriers, using the nanoconfinement effect to precisely regulate the aggregation state of the luminophores, avoiding quenching caused by excessive aggregation, while simultaneously achieving local enrichment of the aggregated luminophores.

Tan *et al.*^140^ used hollow Au–Ag nanoboxes (AuAg NBs) as the core enhancement unit, utilizing the excellent SPR effect of AuAg NBs to enhance the light absorption efficiency of the ECL luminophore, while also taking advantage of their electrocatalytic activity to accelerate the oxidation reaction of co-reactants, increase the generation of free radicals, and enhance the ECL properties of the CoNi nanoflowers/S_2_O_8_^2−^ system.

Ju *et al.*^141^ pioneered a enhanced mechanism by synergistically integrating AIE, RET and thermally activated delayed fluorescence (TADF). This study presents a novel strategy through the design of triethylamine-conjugated polymer dots (TEA-Pdots), which incorporate an AIE-active fluorene derivative as the energy donor and a conventional TADF molecule (DMAC-TRZ) as the acceptor. This system achieves an ECL efficiency of up to 92.6%, significantly outperforming conventional ruthenium complexes and existing organic nanomaterials. This unprecedented ECL performance arises from the synergistic interplay of (i) highly emissive AIE donor, (ii) efficient exciton harvesting *via* TADF in the acceptor, (iii) facilitated dual intramolecular electron transfer enabled by the embedded tertiary amines, and (iv) aggregation-induced RET (ARET) effect within the nanostructure.

## 4 Enhanced-ECL based application

Enhanced ECL, enabled by innovative material design, mechanism optimization, and spatial regulation, has emerged as a powerful tool in bioanalysis and bioimaging. Its inherent advantages of near-zero background, high spatiotemporal resolution, and electrochemical controllability address critical limitations of traditional optical and electrochemical methods, driving breakthroughs in single-molecule detection, single-cell analysis, and disease diagnostics.^11,51,55^

### 4.1 Enhanced-ECL for bioanalysis

High sensitivity is a critical criterion for accurately and precisely quantifying target analytes in bioanalysis.^142–144^ Signal enhancement is imperative to increase sensitivity of advanced ECL devices for expediting their promising applications in bioanalysis, such as protein sensing, and genosensing.^71,145,146^

#### 4.1.1 Protein sensing

Protein sensing is a technology that leverages the specific interactions between biological recognition elements, such as antibodies, aptamers, and enzymes, with target proteins.^147–149^ This process, when combined with detection techniques like ECL, facilitates qualitative identification, quantitative detection, and activity analysis of proteins, particularly biomarkers.

Many works have applied enhanced-ECL to meet the requirement of sensitive detection. Considering that the early Ru(ii)-based ECL systems in the homogeneous phase had limited luminescence efficiency, Yuan *et al.* reported a self-enhanced method that regulates the distance between the luminophore and co-reactant for protein detection.^150^ Using [Ru(bpy)_3_]^2+^ as ECL reactant ligand and ethylenediamine (EDA) as corresponding co-reactant ligand into Zn^2+^ metal node, a dual-ligand Ru(ii)-based coordination polymer, Zn–Ru-EDA, was prepared. The relatively close distance between luminophore and co-reactant within one molecule resulting in less energy loss, enabled high ECL emission. Additionally, the Zn–Ru-EDA complex features a low anodic ECL potential (1.09 V *vs.* Ag/AgCl). It inhibits oxygen evolution to safeguard electrodes and biomolecules, while weakening background noise for superior sensor stability, reproducibility and practicability. This was applied to the detection of procalcitonin with outstanding selectivity and a detection limit of 0.47 fg mL^−1^.

Liu *et al.* proposed an innovative self-enhanced ratiometric ECL strategy to inversely regulate anodic/cathodic signals, for internal calibration without the need for two independent luminophores.^151^ In this design, a bimetallic dual-ligand ECL luminophore, Ce/Tb-MOF, was synthesized *via* a solvothermal approach, exhibiting distinct anodic and cathodic ECL emissions from a single luminophore. Anodic and cathodic signals are spatially separated by using a specially constructed beacon material, TPrA-β-CD@Au, in which TPrA acts as the anodic co-reactant and β-cyclodextrin (β-CD) functions as the aggregation agent to enhance the anodic signal. Based on this mechanism, a highly sensitive ECL immunosensor was developed for the detection of Pro-gastrin-releasing peptide.

A novel ECL emitter, Au NCs@3D porous ZrO_2_ hollow nanospheres (Au NCs@ZrO_2_ HNs), was constructed by utilizing micro/nanochannels and pores as 3D confinement structure.^152^ The porous ZrO_2_ HNs could not only immobilize abundant Au NCs to restrict the free ligand molecular motion of Au NCs, but also facilitate the recombination of holes and electrons of Au NCs during optical processes *via* well-matched energy levels, thereby boosting ECL efficiency. As a result, trace determination of insulin-like growth factor 1 with a detection limit of 0.36 fg mL^−1^ was achieved. However, the inherent poor conductivity of these porous materials somewhat curtails their potential for further enhancement of ECL intensity.^13^

#### 4.1.2 Genosensing

Genosensing utilizes the specific hybridization of nucleic acid probes with target nucleic acid sequences (miRNA, DNA, viral nucleic acids, pathogenic genes, *etc.*), combined with detection techniques such as ECL, to achieve qualitative and quantitative detection and sequence analysis of nucleic acid molecules.^153–156^ Its core applications include genetic diagnosis, genetic disease screening, virus detection, and early warning of tumors.

Plasmonic-based ECL biosensors were the most used strategy in genosensing because the hybridization or conformation change of nucleic acids can regulate the distance between ECL emitters and plasmonic structures. Au nanoparticles are the most used material. To enhance the SPR, researchers generally control the morphology of the noble metals. Gold nanotriangles,^157^ gold nanodendrites,^158^ and nanobipyramids^159^ have been designed to enhance the ECL signal by the tip amplification effect. Utilizing the aforementioned morphological regulation strategies, a series of highly sensitive ECL sensors have been constructed, capable of achieving specific and highly sensitive detection of various RNA. In addition to controlling the morphology of single metals, bimetallic nanostructures also provide a more flexible approach for the fine-tuning of SPR properties. A typical example is the Ag@Au core–shell bimetallic nanostructure, which significantly enhances SPR performance and has been successfully used to construct a microRNA-21 sensor.^160^ Varying the concentration of the initial Ag seed and Au source allowed the size of the Ag core and Au shell to be altered by stepwise galvanic replacement and chemical reduction. The absorption bands of the resultant Ag@Au core–shell nanostructures closely overlapped with the ECL emission spectra of quantum dots, resulting in an effective SPR-enhanced ECL emission. Apart from the noble metal monomers, when two or more nanoparticles are coupled within an effective distance, a stronger local electromagnetic field enhancement will be generated, which in turn has a more significant amplification effect on the ECL signal.^161,162^ In addition, non-metals are also used for enhancement effects, resulting in high-performance sensing.^163^

### 4.2 Enhanced-ECL for imaging

ECL imaging, leveraging the advantages of precise and controllable electrochemistry, high optical sensitivity, and low background interference, has emerged as a pivotal technology for visualizing biosensing, micro-nano interfaces, biological entities, single particles, and bubble reactions.^27,112,135,164–167^ In the realm of ECL imaging research, numerous strategies have been employed to enhance sensitivity and resolution. These include utilizing emitters with superior ECL efficiency, adjusting the reaction potential, elevating the concentration of the emitter or co-reactant, integrating high-resolution single-photon detection devices, and synergizing advanced microscopy with computational techniques for super-resolution reconstruction. While these approaches are instrumental in augmenting imaging performance, they predominantly focus on concentration optimization, device refinement, or hardware enhancement, rather than emphasizing signal amplification from the foundational perspectives of reaction and electron transfer kinetics.

**Fig. 12:**
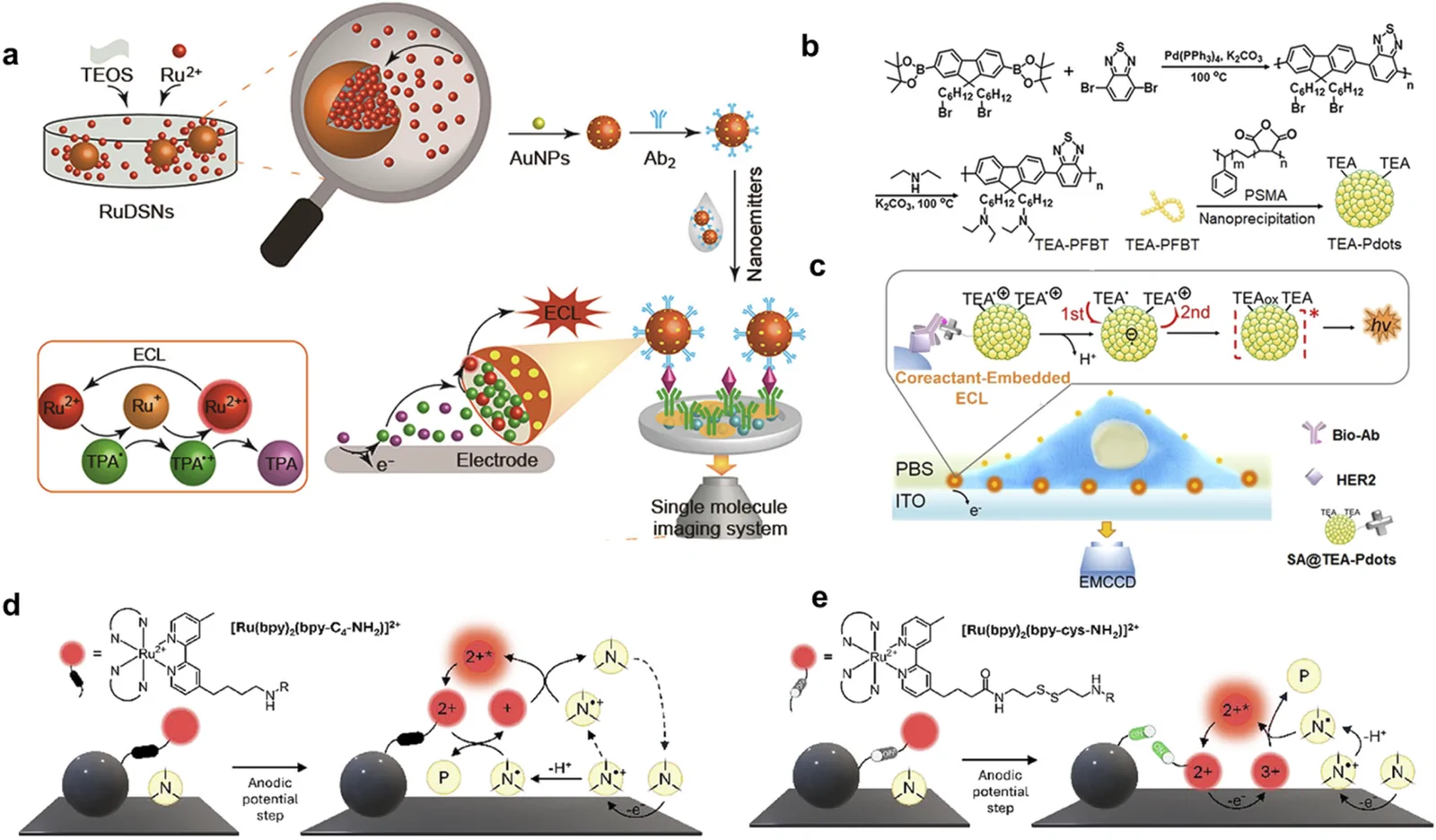
(a) Schematic illustration for single-protein-molecule imaging by confinement effects enhanced ECL. Adapted from ref. 168 with permission from the *American Chemical Society*, copyright 2021. (b) Synthetic route of TEA-PFBT and preparation of TEA-Pdots. (c) Schematic illustration of dual intramolecular electron transfer of TEA-Pdots for ECL microimaging of HER2 on single cells. Adapted from ref. 170 with permission from John Wiley and Sons, copyright 2020. Representation of a conventional bead-based immunoassay-like system in a TPrA solution of (d) [Ru(bpy)_2_(bpy-C_4_–NH_2_)]^2+^ (Ru(C_4_)@Bead) and (e) [Ru(bpy)_2_(bpy-cys-NH_2_)]^2+^ (Ru(S–S)@Bead). Adapted from ref. 52 with permission from the *American Chemical Society*, copyright 2025.

Confinement effects, achieved by incorporating luminophores into microporous or narrow channel structures, have been utilized to enhance the ECL signal. Liu *et al.* loaded [Ru(bpy)_3_]^2+^ onto silica microspheres as a substrate and modified them with gold nanoparticles to achieve ECL enhancement (Fig. 12a).^168^ The porous structure of the silica-based carrier confines [Ru(bpy)_3_]^2+^ on the nanosurface, restricting ECL emission to a local region near the electrode and preventing signal diffusion; the modification with gold nanoparticles improves the conductivity of the carrier, accelerates electron transfer between the electrode and [Ru(bpy)_3_]^2+^, and increases the specific surface area to enrich the co-reactant TPrA, enhancing the probability of reactant collisions. This system, by surface confinement, reduced the ECL background compared to fluorescence imaging, increased the signal-to-noise ratio (S/N) by 17 times, and achieved, for the first time, single-molecule ECL imaging of protein on ITO electrodes and cell membranes. In a similar strategy, leveraging nanoconfinement effects, the [Ru(bpy)_3_]^2+^-embedded porous MOF were designed and exhibited superior ECL performance, including boosted ECL intensity, long-term stability and nearly background-free measurements. As a result, they captured the dynamic trajectories of individual molecules on the cell membrane, and effectively differentiated the heterogeneity of velocity and movement direction among protein individuals in different regions.^169^ In addition, ECL imaging combined with a nanoconfinement enhancement strategy was used to achieve direct visualization of confinement effects within nanoreactors.^64^ It provided an intuitive understanding of how spatial constraints regulate the catalytic performance of nanoreactors: a confined environment can alter the physicochemical properties of reactants and their adsorption-diffusion behavior, thereby enhancing both catalytic activity and selectivity. These findings align with established knowledge in the field of confined catalysis. Ultimately, the nanoconfinement effects on ECL signals are stable even in complex biological conditions, offering an excellent route for single biomolecule imaging. This research not only validates the utility of ECL-enhanced imaging technology for nanocatalysis but also offers an effective visualization method to optimize nanoreactor performance, further encouraging the application and development of ECL imaging technology in catalytic characterization.

Self-enhanced ECL refers to a design that incorporates an ECL luminophore and its corresponding co-reactant within a single unified ECL functional unit. Ju and co-workers introduced two tertiary amine (TEA) groups to the side chain of polymer unit (Fig. 12b and c), which led to a co-reactant-embedded ECL system of Pdots (TEA-Pdots) for self-enhanced ECL imaging.^170^ This design significantly shortens the electron transfer distance between the emitter and the co-reactant intermediates through an intramolecular electron transfer mechanism, thereby overcoming the drawbacks of traditional ECL systems, such as strong dependence on external co-reactants and susceptibility of reaction intermediates to damage. The ECL efficiency of TEA-Pdots was 4 times higher than that of [Ru(bpy)_3_]^2+^/TEA at equivalent mole of emitter and TEA. Based on this ECL enhancement strategy, an ECL imaging biosensor array was developed, achieving high-sensitivity detection of membrane proteins on single living cells.

Recently, stimulus-responsive luminophore-mediated mechanism-switching was proposed for enhanced ECL imaging. To address the limitations of conventional magnetic bead ECL immunoassays, such as restricted sensitivity and high consumption of co-reactants, a [Ru(bpy)_3_]^2+^-derivative luminophore containing a cleavable disulfide bond was designed.^52^ Using electrochemical stimulation as a trigger, efficient switching of the ECL reaction mechanism was achieved, resulting in significantly enhanced signals (Fig. 12d and e). This study combined ECL microscopy imaging, capturing the ECL signal distribution under two reaction mechanisms with ECLM. It was clearly observed that the solution background luminescence intensity in the homogeneous reaction was 208 times that of the traditional heterogeneous system, and the luminescence intensity showed a distinct gradient distribution correlated with the density of magnetic beads. In simulated clinical detection, this enhancement strategy doubled the detection sensitivity for SARS-CoV-2 spike protein, providing a new approach for optimizing the sensitivity of commercial ECL biosensors, enriching the types of ECL enhancement strategies, and expanding their application in clinical immunoassay imaging.

## 5 Conclusions and outlook

This perspective aims to provide a systematic and in-depth understanding of enhanced ECL by examining the advanced characterization and simulation techniques for ECL mechanism interpretation, emerging ECL enhancement pathways, and their cutting-edge applications in bioanalysis and bioimaging. The overall objective is to offer targeted guidance for the rational design and further development of high-performance ECL sensing and imaging systems.

### 5.1 Generalized design of media structures

Despite the great achievements of enhanced ECL systems in ultrasensitive biomolecule detection, single-cell analysis and dynamic biological tracking, several key bottlenecks still restrict their further development and clinical translation. Current enhanced ECL systems are severely restricted by the over-reliance on specific luminophores, including ruthenium(ii) complexes, quantum dots, and MOFs, which greatly limits the universal applicability of ECL platforms for diverse luminescent materials. To address this limitation, it is essential to develop universal, water-soluble, and biocompatible redox mediators that are suitable for various types of luminophores. This strategy can effectively break the material-specific dependence of conventional ECL systems and significantly expand the general applicability of enhanced ECL analytical platforms.

### 5.2 Improvement in efficiency

Generally, ECL pathways face prominent efficiency bottlenecks originating from the poor controllability of key cyclic reaction steps. The unsynchronized rates of radical regeneration and electron transfer seriously hinder ECL signal amplification and restrict the further improvement of detection sensitivity. The corresponding solution is to combine density functional theory (DFT) calculations with *in situ* spectroscopic characterization techniques to establish a quantitative correlation model among key cycle steps, kinetic parameters, and ECL enhancement efficiency. Such models enable precise kinetic matching between radical regeneration and electron transfer, thereby achieving highly efficient ECL signal amplification. In addition, standardizing ECL quantum efficiency measurements across highly diverse and heterogeneous luminescent platforms is important, as historical differences in cell geometries, electrode materials, and mass transport regimes have led to poorly comparable efficiency data. By introducing absolute quantum yield benchmarks or universally accepted reference systems under strictly defined diffusion/convection regimes, the community can achieve precise kinetic matching between radical regeneration and electron transfer, thereby maximizing highly efficient ECL signal amplification.

### 5.3 Precise control of time-scale

Current enhanced ECL techniques suffer from a dynamic mismatch between charge transfer, radical generation, and excited-state evolution across picosecond-to-millisecond time scales. This leads to incomplete reaction utilization and inevitable ECL signal loss, which restricts stable and high-efficiency luminescence output. Because macroscopic ECL intensity is a convoluted flux of transport and chemical cascades, future work should combine *in situ* electrochemistry with ultrafast spectroscopy to quantitatively analyze multi-timescale kinetic behaviors and establish a correlation model between time-scale parameters and ECL enhancement performance. By tracking the early-stage formation of intermediate radicals alongside the subsequent photon emission flux, mathematical models can be constructed to directly map photon emission rates back to specific interfacial electron transfer rates. In addition, expanding time-regulation systems from traditional carbon nitride and quantum dot materials to diverse biocompatible luminophores *via* trap-state engineering and interface charge optimization can realize precise time-scale matching and kinetic synergy, greatly improving the controllability of ECL reaction processes.

### 5.4 Mechanism visualization and quantitative prediction

The insufficient integration of ECL with advanced characterization and analytical technologies results in the lack of visualized and quantitative analysis of microscopic ECL reaction mechanisms, creating a gap between microscale reaction dynamics and macroscale ECL detection performance. The grand challenge here lies in quantitatively mapping the transient spatial–temporal distributions of short-lived radicals within the nanoscale interface. In response, multi-modal characterization technologies including ECL self-interference spectroscopy, electrochemical microscopies, single-molecule spectroscopy, and EC-MS should be comprehensively integrated to precisely capture the dynamic processes of radical generation and electron transfer. Specifically, leveraging high-resolution electrochemical microscopies (*e.g.*, SECCM) alongside single-molecule imaging will allow researchers to reconstruct the localized concentrations and spatial concentration gradients of highly reactive intermediates. Furthermore, the future combination of miniaturized wireless detection devices, and multi-modal imaging technologies (fluorescence, Raman, magnetic resonance imaging) will further improve the quantitative accuracy, portability, and comprehensive analytical performance of ECL systems.

It should be emphasized that in current ECL imaging research, simulation work that truly focuses on ‘revealing reaction mechanisms’ is still in the minority. Most simulations remain at the level of supporting experimental phenomena or validating hypotheses, and their depth heavily depends on model settings and parameter choices. Crucially, intracellular environments or complex serum matrices introduce severe electrode fouling and overlapping redox backgrounds, which often act as a “black box” that corrupts conventional analysis. To develop ECL simulation from a validation tool to a mechanism analysis tool, artificial intelligence (AI) algorithms can be employed to mine complex kinetic data. By deploying physics-constrained algorithms (*e.g.*, Physics-Informed Neural Networks), AI can disentangle the true ECL mechanistic pathways from biological noise, predict ECL enhancement behaviors, and realize intelligent inversion and quantitative interpretation of microscopic reaction mechanisms, which greatly compensates for the limitations of traditional experimental characterization.

## Author contributions

N. S. defined the scope and the structure of the review article. All the authors prepared the manuscript by writing the initial draft, drawing or selecting the illustrations, reviewing and editing.

## Conflicts of interest

There are no conflicts to declare.

## Data Availability

No primary research results, software or code have been included and no new data were generated or analysed as part of this review.
